# Rapid increases in soil pH solubilise organic matter, dramatically increase denitrification potential and strongly stimulate microorganisms from the *Firmicutes* phylum

**DOI:** 10.7717/peerj.6090

**Published:** 2018-12-12

**Authors:** Craig R. Anderson, Michelle E. Peterson, Rebekah A. Frampton, Simon R. Bulman, Sandi Keenan, Denis Curtin

**Affiliations:** The New Zealand Institute for Plant & Food Research Limited, Lincoln Campus, Christchurch, New Zealand

**Keywords:** KOH, Denitrification, Silt-loam soil, N_2_O emissions, Denitrifying bacterial isolates, Clostridia, *Bacillus*

## Abstract

Rapid and transient changes in pH frequently occur in soil, impacting dissolved organic matter (DOM) and other chemical attributes such as redox and oxygen conditions. Although we have detailed knowledge on microbial adaptation to long-term pH changes, little is known about the response of soil microbial communities to rapid pH change, nor how excess DOM might affect key aspects of microbial N processing. We used potassium hydroxide (KOH) to induce a range of soil pH changes likely to be observed after livestock urine or urea fertilizer application to soil. We also focus on nitrate reductive processes by incubating microcosms under anaerobic conditions for up to 48 h. Soil pH was elevated from 4.7 to 6.7, 8.3 or 8.8, and up to 240-fold higher DOM was mobilized by KOH compared to the controls. This increased microbial metabolism but there was no correlation between DOM concentrations and CO_2_ respiration nor N-metabolism rates. Microbial communities became dominated by *Firmicutes* bacteria within 16 h, while few changes were observed in the fungal communities. Changes in N-biogeochemistry were rapid and denitrification enzyme activity (DEA) increased up to 25-fold with the highest rates occurring in microcosms at pH 8.3 that had been incubated for 24-hour prior to measuring DEA. Nitrous oxide reductase was inactive in the pH 4.7 controls but at pH 8.3 the reduction rates exceeded 3,000 ng N_2_–N g^−1^ h^−1^ in the presence of native DOM. Evidence for dissimilatory nitrate reduction to ammonium and/or organic matter mineralisation was observed with ammonium increasing to concentrations up to 10 times the original native soil concentrations while significant concentrations of nitrate were utilised. Pure isolates from the microcosms were dominated by *Bacillus* spp. and exhibited varying nitrate reductive potential.

## Introduction

Soil pH has a strong influence over soil processes such as N-cycling as it impacts soil chemistry, physics and biology. Denitrification is an anaerobic stepwise enzymatic process whereby nitrate (NO${}_{3}^{-}$) is reduced (via NO${}_{2}^{-}$) to nitric oxide (NO), N_2_O and finally molecular nitrogen (N_2_). Denitrification efficiency is primarily affected by soil pH because pH influences carbon supply and associated metabolisms but also impacts the activity of denitrification enzymes adapted to specific pH conditions and the function of N_2_O reductase (N_2_O-R) (Anderson, Peterson & Curtin, 2017; Baggs, Smales & Bateman, 2010; Bakken et al., 2012a; Curtin, Peterson & Anderson, 2016; Liu et al., 2010b; Morkved, Dorsch & Bakken, 2007; Samad et al., 2016b; Schimel, Bennett & Fierer, 2005; Simek & Cooper, 2002). Dissimilatory reduction of NO${}_{3}^{-}$ to NH${}_{4}^{+}$ (DNRA) is also an anaerobic process that reduces NO${}_{3}^{-}$ and variably contributes to N_2_O emissions depending on carbon availability (Giles et al., 2012; Rutting et al., 2011). Again, pH affects carbon supply, alters its accessibility, and thus influences the microbial response. Under anaerobic conditions there is also a strong interplay between carbon availability and whether or not microbes will utilise fermentation or metabolisms such as NO${}_{3}^{-}$ reduction (Van den Berg et al., 2017a; Van den Berg et al., 2017b). Although there is a reasonable mechanistic understanding of how changing soil pH affects chemistry and physics, the literature is less robust concerning the dynamics of biological response at molecular levels both phylogenetically and functionally.

The long-term effects of pH on microbial community structure and abundance have been studied at local to global scales, but only broad conclusions can be drawn (Fierer & Jackson, 2006; Lauber et al., 2009). For example, at the localised scale in the Rothamsted Hoosefield acid strip, bacterial ‘richness’ has been shown to increase between pH 4 and 8, whereas changes in fungal populations were not pronounced (Rousk et al., 2010a); or within the Rothamsted Park Grass Experiment where 14 of the 37 most abundant soil genera were positively related to soil pH (Zhalnina et al., 2015). Relationships become more tenuous at larger scales with pH known to shape microbial communities, but this seems to be based more on functional genetic diversity than taxonomic classification. For example, at continental scales there is evidence to suggest that Acidobacteria populations have a negative trend as pH increases (>4) but within phyla the relative abundance of Acidobacteria subgroups exhibit opposing trends as pH increases (Lauber et al., 2009).

In contrast to studies describing microbial community response to pH in long-term trials, reports about community response to rapid, short-term pH change are sparse; yet microbial communities in soils are often subjected to dynamic change. For example, rapid pH change after animal urine deposition or in the vicinity of urea fertiliser prills. When urea (CO(NH_2_)_2_) is added to the soil (as fertiliser or urine), it quickly undergoes hydrolysis in the presence of urease enzymes:

\begin{eqnarray*}\mathrm{CO}({\mathrm{NH}}_{2})_{2}+{\mathrm{3H}}_{2}{\mathop{\mathrm{O}\rightarrow \mathrm{2NH}}\nolimits }_{4}^{+}+{\mathrm{2OH}}^{-}+{\mathrm{CO}}_{2}. \end{eqnarray*}

The OH^−^ ions produced during this process cause substantial pH increases, to values >7.5 over the course of a few days, coupled with the pH mediated release of dissolved organic matter (DOM) (Clough et al., 2010; Curtin, Peterson & Anderson, 2016; O’Callaghan et al., 2010). During the first week post urine deposition, pH continues to rise (to values > pH 8), ammonium (NH${}_{4}^{+}$) oxidation to NO${}_{3}^{-}$ (via NO${}_{2}^{-}$) commences, while oxygen concentrations and redox conditions decrease via nitrification reactions and through microbial metabolism (Clough et al., 2010; Hansen, Clough & Elberling, 2014; Nowka, Daims & Spieck, 2015)—suitable conditions for NO${}_{3}^{-}$ reductive processes. A recent study (Anderson, Peterson & Curtin, 2017), using KOH or Ca(OH)_2_ as proxies for NH_4_OH indicates that denitrification response becomes elevated very shortly after pH and DOM increases.

In addition to allowing documentation of soil physicochemical response, urine patches represent a natural laboratory setting for investigating microbial community structural and functional response to rapid pH change. A few studies have reported changes in soil microbial population structures in response to urine (or synthetic urine), but have not investigated the taxonomic profiles of these populations in detail (Rooney et al., 2006; Singh, Nunan & Millard, 2009). In the study of Singh, Nunan & Millard (2009), addition of synthetic urine to soil was not associated with increases in microbial biomass C or N and the relative population structure of fungi did not change. Taxonomic changes observed by O’Callaghan et al. (2010) indicated that *Firmicutes* increased by 38% after urine addition while Proteobacteria decreased (18%), but fine resolution of taxonomic groups contributing to denitrification or any other N-cycling process was not achieved.

Using soil microcosms, we sought to simulate the effects of rapid soil pH change likely to occur under urine patches or around urea prills during the first 48 h post urea deposition. We hypothesised that the microbial population would undergo major structural and physiological change in response to pH, increases in DOM and decreases in oxygen; with N-processing shifting to NO${}_{3}^{-}$ reductive processes. In order to change soil pH and isolate the denitrification processes without adding extra N (or C) via urea, we used potassium hydroxide as an NH_4_OH proxy (see Anderson, Peterson & Curtin, 2017) and utilised anaerobic conditions. After different exposure times to four different pH treatments, the potential denitrification enzyme activity (DEA) of the microbial communities was assayed, amplicon sequencing was used to provide a detailed assessment of the changes in the microbial populations, and a collection of nitrate reducing bacteria were isolated from the microcosms.

## Materials and Methods

All aqueous solutions were prepared using ultrapure water from a MilliQ water system (18 M Ω-cm resistivity) and all chemicals used were ACS reagent grade, unless otherwise stated.

### Soil collection, pH adjustment and DEA assays

The Wakanui silt-loam soil used in this study was sourced from no-till plots in a long-term field trial (12 years) at Lincoln, Canterbury, New Zealand. The basic chemical characteristics of the soil were: pH 5.6; total C, 27 g kg^−1^; total N, 2.4 g kg^−1^; NO_3_-N, 20 mg kg^−1^ and NH_4_-N, 4 mg kg^−1^. Further details about the soil and sampling site can be found in Curtin, Peterson & Anderson (2016) and Anderson, Peterson & Curtin (2017). Soil samples were treated with four rates of KOH (base addition rates of 0, 6.0, 16.0 & 20.0 cmol_c_ kg^−1^ soil). These treatments were selected based on results from previous experiments (Anderson, Peterson & Curtin, 2017) with the 6.0 cmol_c_ kg^−1^ and 16.0 cmol_c_ kg^−1^ treatments representing “low/moderate” and “upper limit” pH increases following animal urine deposition. The 20 cmol_c_ kg^−1^ treatment represented an alkaline pH outlier, where DEA was expected to be minimal (Anderson, Peterson & Curtin, 2017).

A total of 240 soil microcosms were prepared, covering 4 KOH treatments and 5 incubation times. This provided twelve analytical replicate microcosms for each KOH rate ×  incubation time combination; where, four microcosms were designated for soil chemical analysis prior to DEA assays, two were designated for nucleic acid extraction and microbial culturing work (prior to DEA), and the remaining six microcosms were used for DEA assessments (two triplicate DEA assays, with or without acetylene) (Fig. S1).

Microcosms were prepared as described by Anderson, Peterson & Curtin (2017). Briefly, 25 g (dry weight equivalent) soil was placed in 250 mL bottles and KOH was added together with KCl to balance electrical conductivity across treatments. The final solution volume in each microcosm was adjusted to 25 mL. The bottles were evacuated (to −1 atm.) then flushed three times with N_2_ (instrument grade, <0.001% O_2_) over a 30 min period until O_2_ was <0.03%. The microcosms were then incubated at 20 °C on an orbital shaking platform (150 rpm) for 16, 24, 32, 40 or 48 h. After each incubation the headspace was sampled for N_2_O and CO_2_.

The following sampling protocol was followed: for chemical analyses, 5 mL of slurry was collected from four microcosms to determine dissolved CO_2_ (acidified with 2 mL of 2M HCl to dissolve any carbonates). The remaining slurry from these four microcosms was centrifuged (5min at 20,000 rpm), and the supernatants filtered (<0.45 µm) then frozen (−20 °C), pending pH, EC, DOC, DON and NO${}_{3}^{-}$/NH${}_{4}^{+}$ analyses. From a further two microcosms, 1 mL aliquots of slurry were taken for nucleic acid extraction and bacterial colony isolations.

To determine DEA, 5 mL of water containing 50 mg NO_3_-N kg^−1^ (Luo et al., 1996) was added to the remaining six microcosms, and the anaerobic atmosphere was regenerated by evacuation and flushing three times with N_2_. To three of the microcosms a volume of scrubbed (Hyman & Arp, 1987) acetylene (final ratio of 10% v/v) was added by syringe and allowed equilibrate (with shaking) for 10 min before venting these microcosms to atmospheric pressure. No external C-sources such as glucose were added. The microcosms were incubated at 20 °C on a shaking platform (150 rpm) and sampled hourly over a 4 h period to measure headspace N_2_O and CO_2_. Headspace gases removed were replaced with an equivalent volume of N_2_.

At the end of the DEA assay, dissolved CO_2_, pH, EC, DOC, DON and NO${}_{3}^{-}$/NH${}_{4}^{+}$ were measured in the remaining soil slurry samples.

### Chemical analysis and gas chemistry

Analytical methods are described in Anderson, Peterson & Curtin (2017). Briefly, concentrations of N_2_O and CO_2_ were determined on a Shimadzu Corp. GC-17A gas chromatograph and the DEA value (i.e., potential denitrification rate) and respiration rates were calculated from the linear relationship between evolved N_2_O or CO_2_ and time. The extracts were analyzed for pH (ThermoScientific Orion™ AquaPro™ pH combination electrode) and electrical conductivity (Eutech Instruments PC510 conductivity meter). Dissolved organic C was determined using a Total Organic Carbon Analyzer (Shimadzu TOC-V _CSH_, Shimadzu Corp, Japan). Total N was determined by persulfate oxidation, as described by Cabrera & Beare (1993), and organic N was estimated by subtracting mineral N (KCl extracted NH${}_{4}^{+}$ and NO${}_{3}^{-}$ determined using an automated colorimeter) from total N.

### Microbial population profiling via next generation sequencing

A 1 mL aliquot of soil slurry was centrifuged at 14,000 rpm for 5 min. DNA from the resulting pellet was extracted with the MoBio Powersoil DNA kit (Carlsbad, CA). The V3–V4 variable regions of the bacterial 16S rRNA was amplified with the 341f and 785r primer pair (Klindworth et al., 2013). The fungal internal transcribed spacer 1 (ITS1) region was amplified with NSI1a_mod (5′-GATTGAATGGCTTAGTGAGK-3′) and 58A2R (5′-AGTCCTGCGTTCTTCATCGAT-3′), both adapted from (Martin & Rygiewicz, 2005). Primers included the Illumina adapter sequences.

PCR amplifications contained ∼10 ng DNA template, 10 nmol each primer, 1×  mastermix, and 0.5 U KAPA3G polymerase (Merck, Auckland, New Zealand), in a final volume of 20 µl. Reactions were performed in duplicate. Cycling parameters were 94 °C for 2 min; 30 cycles of 95 °C for 30 s, 50 °C for 30 s, 72 °C for 30 s. Duplicate reactions were combined and purified with AMPure XP beads (Agencourt, Beckman Coulter Life Sciences). Purified amplicons were quantified by gel electrophoresis and UV absorbance (NanoDrop ND-1000). Amplicons were 2 ×300 bp paired end sequenced on an Illumina MiSeq platform (New Zealand Genomics Limited, Auckland).

### Sequence processing and statistical analysis

USEARCH v8.0.1517 (Edgar, 2013) was used to merge the paired end reads, filter chimeric sequences and cluster sequences at 97% similarity. An expected error of 1.0 was used for filtering. Singleton reads were discarded. The bacterial 16S OTUs were identified using the RDP Naïve Bayesian Classifier implemented in USEARCH against Greengenes (version 13_8) and fungal ITS OTUs against the UNITE reference dataset (Version 6, 04∕07∕2014, downloaded on 08∕07∕2014) (http://www2.dpes.gu.se/project/unite/UNITE) (Koljalg et al., 2013). Biom (OTU) tables were produced using biom-format (http://biom-format.org/) (McDonald et al., 2012) in USEARCH with rarefication performed in phyloseq.

The phyloseq (McMurdie & Holmes, 2013) and ggplot2 (Wickham, 2009) packages within R (R Core Team, 2017) were used for the analysis and visualisation of data at phyla level. OTU tables were also analysed using Primer 7 with PERMANOVA add-on (Primer-E Ltd, Plymouth, UK). Both rarefied and non-rarefied data was analysed based on the work of McMurdie and Holmes (McMurdie & Holmes, 2014). Where data was not rarefied, samples were standardised by total and no statistical inferences were made regarding differentially abundant species (OTUs), rather our conclusions were based on assessing broader scale relative changes in the microbial communities only. Relationships among microbial community profiles based on Bray–Curtis similarity matrices were graphed using unconstrained non-metric multidimensional scaling (nMDS) ordinations using 250 restarts along with cluster analysis. Relationships observed among all OTUs were then statistically tested using 2-factor permutational ANOVA within the Primer 7 package (PERMANOVA) with estimated components of variation being a standard output of the analysis. Data was untransformed unless otherwise stated, whereupon log(*X* + 1) transformations were applied.

To understand gradients and group structures across treatments, matrix plots of standardised data were prepared using a reduced sample set of 20 OTUs, with the OTUs retained having the greatest contribution to total counts for the individual samples compared at each pH value, or in the case of fungi, across all pH values. Using reduced sample sets allows simplification of the matrix plots by removing those organisms accounting for a negligible proportion of the total number of OTUs. Samples in the matrix plots were clustered using Bray Curtis similarity (based on all OTUs) while the 20 OTUs presented were clustered according to similarity based on an index of association across samples tested.

### Isolation of bacteria, N-use characterisation

#### Isolation of nitrate reducing bacteria

Previous research by O’Callaghan et al. (2010) suggested that Firmicutes increased by substantial amounts in conditions similar to those we investigated. Although only a small percentage of soil bacterial diversity can be cultured, it is relatively straightforward to culture members of the Firmicutes (among others); hence, in order to gain some appreciation for the N-processing capabilities of culturable bacteria from these microcosms, we attempted to isolate NO${}_{3}^{-}$ reducers.

Ten µL sample of slurry from each pH adjusted microcosm was serially diluted (10 µL into 1 mL followed by three dilutions of 10 µL into 100 µL) and plated onto TSB or 1/10 diluted TSB containing KNO_3_ (30g L^−1^ + 0.5 g L^−1^ KNO_3_). Plates were incubated under anaerobic conditions (Whitney jars with gas packs) at 24 °C for 2 to 6 days.

Representative colonies of different morphology were selected and re-streaked onto the same media and grown under anaerobic conditions at 24 °C. Isolates were stored in 20% glycerol at −80 °C, with a selection of isolates identified by amplification and Sanger sequencing of the 16S ribosomal RNA gene using the 27F and 1492R primers (Anderson et al., 2009).

#### Nitrate utilisation by bacterial isolates

Cells were grown aerobically overnight in TSB with KNO_3_. 20 mL cultures were then initiated with 1/100 dilutions of the overnight cultures, and grown anaerobically (O_2_ replaced with N_2_) at 24 °C. After 48 h, gas samples were extracted with a gas-tight Hamilton syringe and analysed for the presence of N_2_O and CO_2_ by gas chromatography (as outlined above) and 10 mL samples of the bacterial cultures were removed for NO${}_{3}^{-}$ and NH${}_{4}^{+}$ analysis (as outlined above).

## Results

### Chemical characterisation of pH amended soils prior to DEA assays

After 48 h of anaerobic incubation, soil slurries with additions of 0, 6, 16 and 20 cmol_c_ kg^−1^ KOH had average pH values of 4.7, 6.7, 8.3 and 8.8 respectively. During the 48 h incubation EC reached ∼6.4 mS cm^−1^ in all microcosms except the pH 6.7 microcosms which reached ∼5.7 mS cm^−1^ (Table 1). DOC in the control microcosms increased from 37 to 63 mg kg^−1^ during the 48 h incubation. In the pH 6.7, 8.3 and 8.8 microcosms DOC increased ∼11, 185 and 240-fold respectively in response to KOH addition, with the majority of that change (>75%) occurring during the first 16 h of incubation. During the 48 h incubation DON in the control microcosms increased from 2.4 to 7.4 mg kg^−1^, while in the pH 6.7, 8.3 and 8.8 microcosms DON increased ∼9, 140 and 223-fold in response to KOH addition, respectively (Table 1). There was a strong correlation between the amounts of DOC and DON solubilised at each pH value, irrespective of incubation time (*R*^2^ = 0.98) (Fig. S2).

**Table 1:** N-chemistry trends, cumulative respired CO2 and EC data for each pH treatment. Sampling occurred at the end of each incubation period and prior to DEA assays with 4 replicate microcosms for each pH treatment and incubation time.

	**Pre-DEA Incubation time (h)**	**pH** (KOH addition—cmol_*c*_ kg^−1^)							
		**4.7 [0]**		**6.7 [6]**		**8.3 [16]**		**8.8 [20]**	
		**Ave.**	**SD**	**Ave.**	**SD**	**Ave.**	**SD**	**Ave.**	**SD**
**NH**${}_{4}^{+}$	**16**	1.8	0.1	5.2	0.2	20.9	0.7	30.5	1.4
(mg kg^−1^)	**24**	2.5	0.2	8.2	0.4	27.4	1.4	31.0	0.7
*4*^a^	**32**	3.0	0.2	10.1	0.1	35.5	3.0	32.1	2.0
	**40**	3.6	0.3	11.9	0.2	41.9	2.3	35.2	0.9
	**48**	5.0	0.6	14.4	0.9	45.2	3.0	50.4	1.0
**NO**${}_{3}^{-}$	**16**	5.4	1.0	0.2	0.1	13.0	0.3	18.3	0.5
(mg kg^−1^)	**24**	3.94	0.2	0.2	0.0	3.3	0.3	18.5	0.4
*20*^a^	**32**	4.7	0.6	0.3	0.1	4.2	0.5	18.8	1.1
	**40**	0.9	0.3	0.3	0.1	5.0	0.3	16.4	0.3
	**48**	0.2	0.1	0.3	0.1	4.8	0.4	7.4	1.1
**N**_2_**O**	**16**	1,651	75.9	849.3	323.8	11.0	3.0	3.2	4.4
(µg kg^−1^)	**24**	3,587	1,136	10.1	12.9	125.1	22.9	4.3	1.9
	**32**	4,408	236.8	5.4	10.0	0.6	2.0	7.1	4.2
	**40**	5,422	1,748	9.8	15.1	0.8	1.1	9.6	2.6
	**48**	6,810	503.5	13.0	14.4	2.6	6.6	14.0	16.8
**CO**_2_	**16**	8.2	1.0	17.5	0.8	15.4	1.5	13.5	1.6
(mg kg^−1^)	**24**	14.6	4.9	34.4	3.5	23.0	3.7	12.0	1.1
	**32**	25.5	2.3	56.6	14.6	34.0	8.6	8.2	1.1
	**40**	19.6	4.3	43.7	4.9	58.6	3.2	15.8	4.9
	**48**	21.1	1.8	49.3	4.9	76.8	17.5	39.2	14.2
**DOC**	**16**	36.7	4.4	226.9	9.4	4,846	106.0	5,670	800.5
(mg kg^−1^)	**24**	39.5	3.9	232.9	14.0	4,767	322.8	5,996	2,038
	**32**	45.7	4.1	266.5	25.0	4,608	147.6	5,483	1,029
	**40**	55.2	2.6	264.8	12.8	4,543	232.5	5,582	786.0
	**48**	62.9	4.2	301.8	12.5	4,983	145.7	6,493	498.5
**DON**	**16**	3.5	0.7	18.3	1.4	338.3	51.2	440.6	37.3
(mg kg^−1^)	**24**	5.6	0.5	15.8	3.5	323.4	22.4	490.8	57.1
	**32**	6.1	1.0	16.4	2.5	340.5	13.4	542.8	73.6
	**40**	7.3	0.3	18.0	1.4	367.3	41.4	462.8	185.8
	**48**	7.4	1.1	21.5	2.2	335.4	26.2	536.2	97.6
**EC**	**16**	5.0	0.0	4.5	0.1	5.4	0.1	5.8	0.2
(mS cm^1^)	**24**	5.9	0.1	5.2	0.1	6.0	0.2	6.0	0.1
	**32**	6.4	0.0	5.6	0.1	6.2	0.1	6.1	0.1
	**40**	6.4	0.1	5.7	0.1	6.2	0.0	6.2	0.2
	**48**	6.4	0.1	5.7	0.1	6.4	0.2	6.4	0.1

**Notes.**

a Native soil concentration of NH${}_{4}^{+}$ and NO${}_{3}^{-1}$ prior to experiments.

Higher respiration (CO_2_ production) was associated with DOM increases but the amount of DOC (or DON) solubilised via pH change was not a good predictor of respiration. Respiration in the control and pH 6.7 microcosms generally followed an increasing trend for the first 32 h before decreasing (Table 1). The pH 8.3 microcosms exhibited respiration rates that increased throughout the 48 h incubation period (maximum recorded rate of ∼1,600 ng CO_2_-C g^−1^ h^−1^). Respiration in the pH 8.8 microcosms followed an opposing trend, declining slightly during the first 32 h, followed by recovery.

In control microcosms, ammonium (NH${}_{4}^{+}$) increased linearly over 48 h with a slope of 0.09, starting from native soil concentrations of ∼4 mg kg^−1^. The NH${}_{4}^{+}$ profiles in the pH 6.7, and 8.3 microcosms were similar (approximately linear) but with greater slopes of 0.28 and 0.79 respectively (*R*^2^ values of 0.99 and 0.98). In the pH 8.8 microcosms, NH${}_{4}^{+}$ quickly elevated to 30 mg kg^−1^ during the first 16 h of incubation and remaining at that concentration until after 32 h, when a further increase from 30 to 50 mg kg^−1^ occurred by 48 h (Table 1). The native soil nitrate (NO${}_{3}^{-}$) concentration was ∼20 mg kg^−1^. This NO${}_{3}^{-}$ was almost completely reduced after 32–40 h incubation in the control, pH 6.7 and pH 8.3 microcosms (Table 1). In the pH 8.8 microcosms, NO${}_{3}^{-}$ remained at ∼20 mg kg^−1^ until after 32 h incubation, dropping to ∼6 mg kg^−1^ during the following 16 h.

The control microcosms had the highest concentrations of N_2_O in the headspace, increasing from ∼1,650 to 6,800 µg kg^−1^ between 16 and 48 h of incubation (Table 1). The pH 6.7 microcosms had ∼850 µg kg^−1^ of N_2_O in the headspace after 16 h incubation, but by 24 h this had declined to <15 µg kg^−1^. Headspace N_2_O in the pH 8.3 and 8.8 treatments was <15 µg kg ^−1^for all time points except 24 h for the pH 8.3 treatment (125 µg N_2_O kg^−1^ soil).

### DEA assays

**Figure 1:**
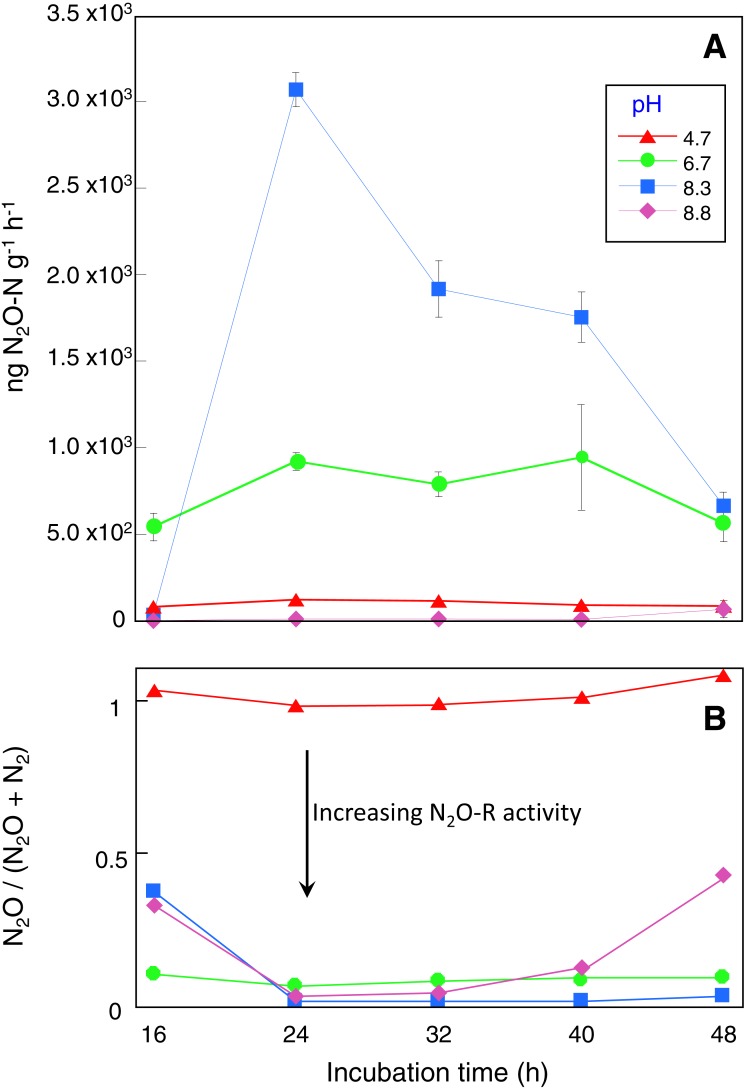
Denitrification enzyme activity assay results. Sampling occurred at the end of each incubation period and prior to DEA assays with four replicate microcosms for each pH treatment and incubation time.

Microcosms incubated for 16 h had the lowest DEA rates with 80, 540, 34 and 0.2 ng N_2_O g^−1^ h ^−1^ recorded for the control, pH 6.7, 8.3 and 8.8 treatments respectively (Fig. 1A). DEA remained low in the control microcosms with a maximum DEA rate of only 121 ng N_2_O g^−1^ h^−1^ after 24 h incubation, declining to 85 ng N_2_O g^−1^ h^−1^ in microcosms incubated for 48 h (Fig. 1A). In contrast, after 24 h incubation, DEA rates in the pH 6.7 and 8.3 microcosms were 8 and 25-fold higher respectively with a maximum of ∼3,000 ng N_2_O g^−1^ h^−1^ produced (Fig. 1A). DEA rates remained in the vicinity of 900 and 2,000 ng N_2_O g^−1^ h^−1^ for the pH 6.7 and 8.3 treatments, respectively, for microcosms incubated up to 40 h. DEA rates greater than those in the control were not observed in the pH 8.8 treatment until microcosms were incubated for at least 48 h (∼65 ng N_2_O g^−1^ h^−1^).

Replicate microcosms without acetylene added were used to assess N_2_O-R activity during the DEA assays based on the N_2_O/(N_2_O + N_2_) ratio. These showed that N_2_O reduction was absent in the control microcosms while in the pH 6.7, 8.3 and 8.8 treatments between 56 and 100% of the N_2_O produced was reduced to N_2_ depending on incubation time (Fig. 1B). Near complete N_2_O reduction was observed in the pH 8.3 treatment for microcosms incubated longer than 24 h. Depending on pH treatment and incubation time, N_2_O production (and reduction) profiles during the DEA assays were mirrored by decreases in NO${}_{3}^{-}$ with an estimated 30 to 80% of the available NO${}_{3}^{-}$ reduced. In the control and pH 6.7 microcosms there were no differences in the NH${}_{4}^{+}$ before and after the 4-hour DEA assay period, however decreases of up to 9 mg kg^−1^ occurred in the pH 8.3 microcosms after 32 h incubation and in the 8.8 microcosms after all incubation times (Table ST1).

*T*-tests comparing CO_2_ respiration indicated that acetylene addition depressed microbial activity, but only in the pH 8.3 and 8.8 treatments (*P* < 0.05). It is acknowledged that lower soil respiration in the presence of acetylene will reflect both the absence of any CO_2_ derived from metabolic N_2_O reduction but possibly also general impediment of other anaerobic metabolisms. It is unlikely that acetylene would have served as dominant carbon source during the 4 h DEA incubation given the excess DOC available and time required to adapt to using acetylene (Felber et al., 2012; Groffman et al., 2006). There is evidence to suggest that DOC and DON declined during the DEA assay period, especially in the pH 8.3 and 8.8 treatments but the results were highly variable (Table ST1).

### Microbial community adaptation to pH treatments during 48 h incubation

**Figure 2:**
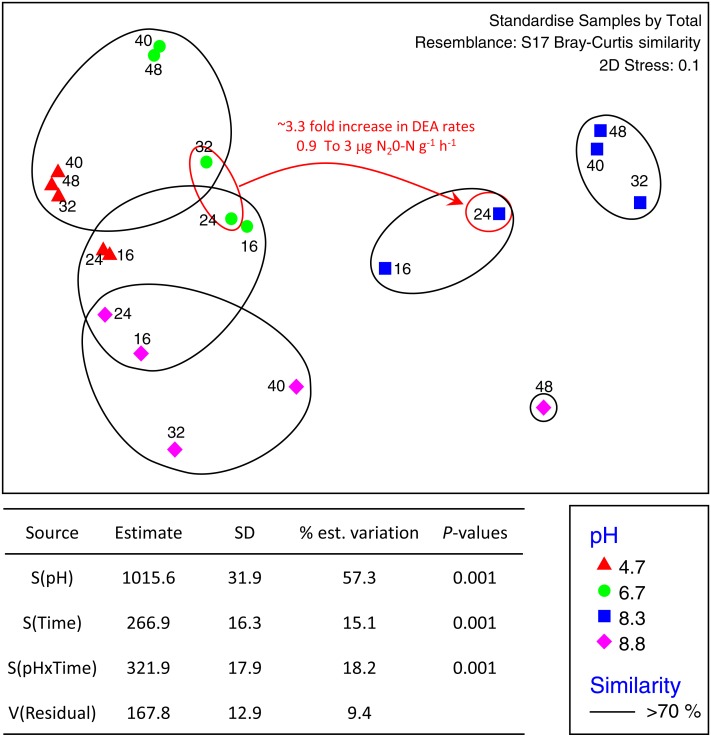
nMDS ordination of bacterial OTUs identified using Illumina sequencing of the 16s rRNA gene. OTU tables were compared using a Bray–Curtis similarity matrix with data standardised by total. The nMDS represents an unconstrained ordination of the Bray Curtis similarity measures from 250 restarts allowing examination of the broad relationships between microbial communities from each pH treatment and incubation time point. The 2D stress value of 0.1 indicates a good ordination with a low chance of a misleading interpretation. Bacterial communities among samples enclosed by black rings share >70% similarity, highlighting the level of similarity across both pH treatment and time. The estimated components of variation and interaction between sources of variation calculated via PERMANOVA are presented in the table below.

**Figure 3:**
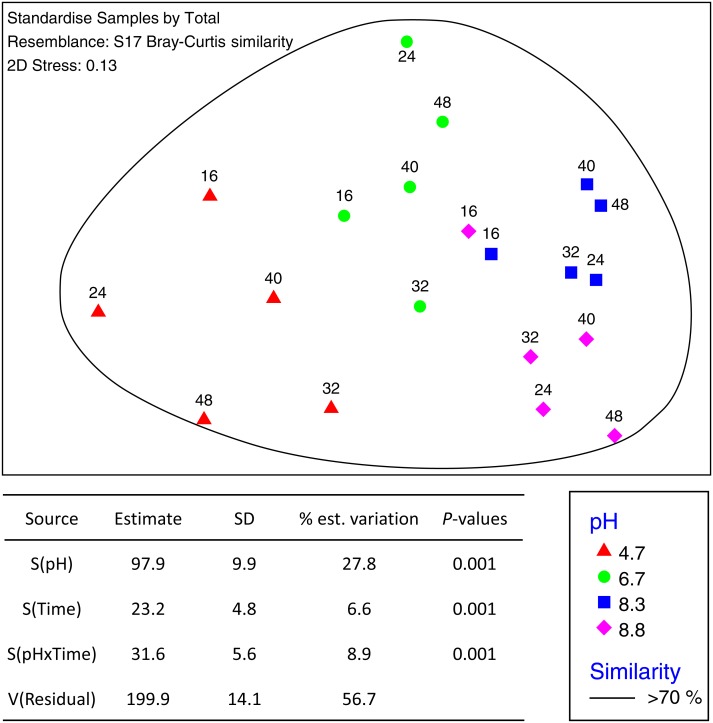
MDS ordination of fungal OTUs identified using Illumina sequencing of the fungal ITS1 region. OTU tables were compared using a Bray–Curtis similarity matrix with data first log(*X* + 1) normalized and standardised by total. The nMDS represents an unconstrained ordination of the Bray Curtis similarity measures from 250 restarts allowing examination of the broad relationships between microbial communities from each pH treatment and incubation time point. The 2D stress value of 0.14 indicates a good ordination with a moderate chance of a misleading interpretation. Fungal communities in samples enclosed by the black ring share >70% similarity, highlighting the high level of similarity in fungal communities with pH treatment and across time. The estimated components of variation and interaction between sources of variation are presented in the table below.

The total number of OTUs identified across all treatments and incubation times was 2,258 for fungi and 6,429 for bacteria with an average of 84,000 reads per sample. Sequences from this Targeted Locus Study (LTS) project has been deposited at DDBJ/EMBL/GenBank under the accession KCDA00000000. The version described in this paper is the first version, KCDA01000000. Fungal ITS1 sequences have been deposited at DDBJ/EMBL/GenBank under the accession numbers MH624180 –MH625694. Two factor tests using PERMANOVA [pH ×  incubation time] for all OTUs supported the nMDS observations indicating significant interactions between time and pH (*P* = 0.001 for both bacteria and fungi) (Fig. 2). Approximately ∼90% of the total variation was explained for the bacterial relationships whereas only ∼43% was explained for the fungi. Of the variation explained for the bacteria, up to 60% was attributed to pH, a further 15% to incubation time, and 18% was attributed to the interaction between the two factors. The corresponding values for the fungal communities were 28% of variation attributed to pH, 6.6% to incubation time and 8.9% to the interaction between the two factors (Fig. 3).

**Figure 4:**
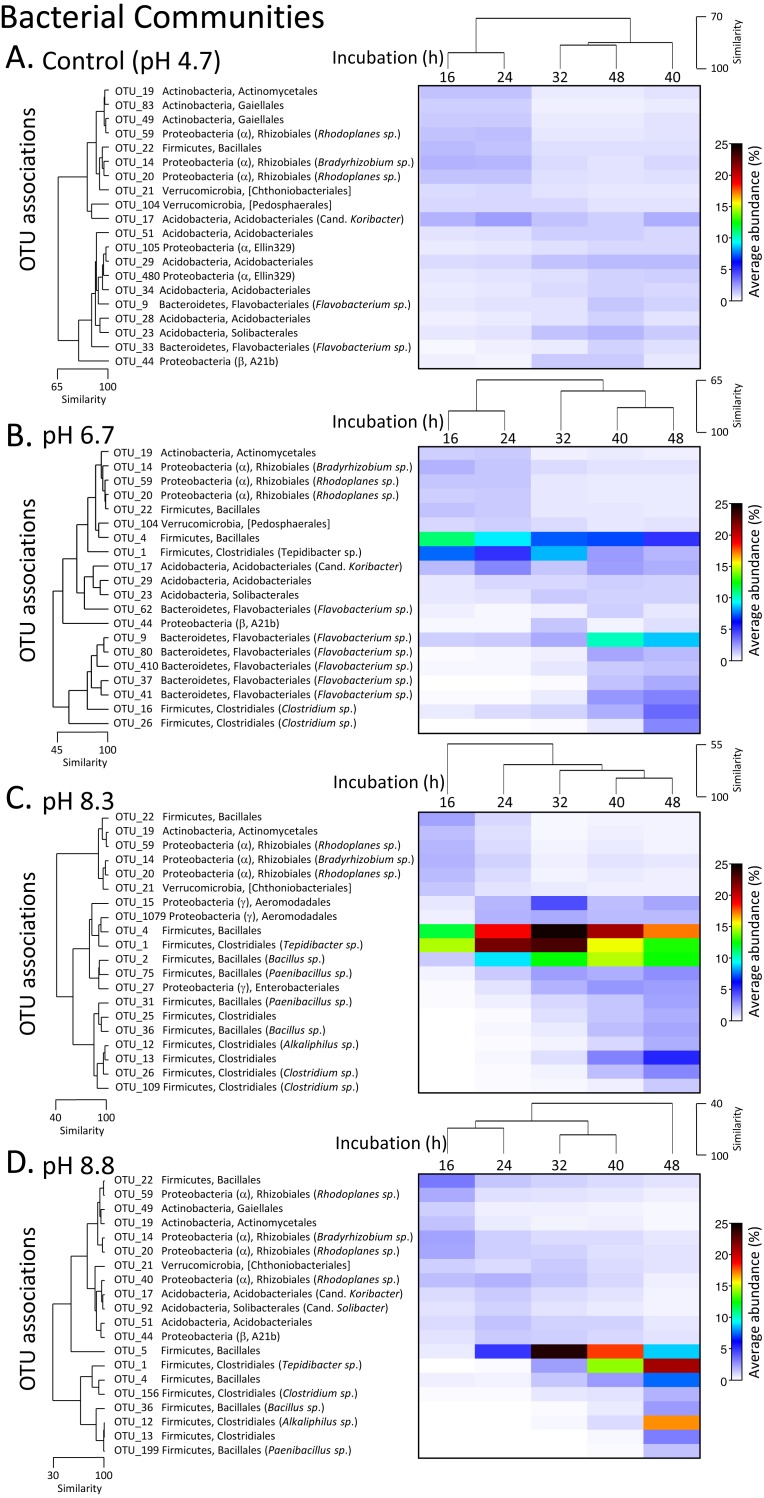
Matrix plots of the relative abundance and clustering of the 20 bacterial OTUs with the highest contribution to total sequence counts across incubation times for each pH treatment. Control microcosms (A), pH 6.7 (B), pH 8.3 (C) and pH 8.8 (D). Samples for each pH treatment have been clustered via Bray Curtis similarity measures representing the entire set of OTUs while the 20 OTUs selected to represent major changes in each treatment have been clustered via an index of association. Similarity measures have been provided to enable comparison of changes as incubation time (h) increases. Average ‘abundance’ should be treated as a relative indication only. These ‘abundances’ represent data that is non-transformed and standardised-by-total.

The phylum level bacterial profile from the control (pH 4.7) microcosms after 16 h incubation was made up of Acidobacteria (∼7%), Actinobacteria (∼12%), Bacteroidetes (∼7%), Proteobacteria (∼20%), Firmicutes (∼5%), Planctomycetes (∼5%) and phyla that had abundances of >5% including Verrucomicrobia, Chloroflexi, Gemmatimonadetes and Armatimonadetes. Up to 35% of OTUs could not be classified (Fig. S3). After 24 h incubation, the bacterial communities were still >85% similar before there was a relative decrease in Actinobacteria coupled with an increase in Acidobacteria and Bacteroidetes (Fig. S3). A decrease in Actinobacteria was also seen over the incubation periods for all three pH modifications along with changes in the proteobacterial populations with almost complete disappearance of OTUs from the order Rhizobiales (Fig. 4, Fig. S3).

After 16 and 24 h incubation, the bacterial profiles in the pH 6.7 microcosms were similar to those in the control microcosms with the exception of a larger proportion of Firmicutes (∼20% versus 5% in the controls) (Fig. S3). The increased representation of Firmicutes was driven by an expansion of OTUs from the orders Bacilliales (OTU_4) and Clostridiales (OTU_1, *Tepidibacter sp.*). These two OTUs then decreased later in the incubation, partially displaced by other *Clostridium spp.* (OTUs 16 and 26) (Fig. 4B). Across the 32 to 48 h incubation period there was a large relative increase in OTUs from the phylum Bacteroidetes, driven by *Flavobacterium spp.* (OTUs 62, 9, 80, 410, 37 and 41) (Fig. 4B).

In the pH 8.3 treatment after 16 h incubation, there was a very high proportion of Firmicutes (∼40%) and a low representation of Actinobacteria and Bacteroidetes compared with the pH 4.7 control microcosms. Across the incubation period, the dominance of Firmicutes increased further, initially driven by the same Bacilliales and Clostridiales OTUs (4 and 1), but at a much higher relative ‘abundance’ than observed in the pH 6.7 treatment. Their expansion coincided with the highest DEA rates, with the average ‘abundance’ of OTU_4 being ∼20% of total at this time and OTU_1 being ∼23% (Fig. 4C, Fig. S3). After 32 h these two dominant OTUs were partially displaced by a cohort of other OTUs from the orders Bacilliales and Clostridiales (OTUs 2, 75, 31, 25, 36, 12, 13, 26 and 109) (Fig. 3). At the genus level these OTUs represented *Bacillus*, *Paenibacillus*, *Clostridium* and *Alkaliphilus* spp. (Fig. 3). Communities in the 40 and 48 h incubations shared the highest level of similarity (Figs. 2, 4C and Fig. S3).

After 16 and 24 h incubation, the bacterial profiles from the pH 8.8 treatment shared >70% similarity with the samples from the control microcosms (Fig. 2). Over the incubation period to 32 h, there was a large expansion in the relative percentage of the Firmicutes, initially driven almost entirely by an increase in OTU_5 from the Bacilliales order (Fig. 4D). By 48 h, this OTU was partially displaced by a group of Bacilliales and Clostridiales OTUs that shared some similarity with those observed in the pH 8.3 treatment (OTUs 1, 4, 156, 36, 12, 13, and 199). These OTUs included the same genus level representatives—*Bacillus*, *Paenibacillus*, *Clostridium* and *Alkaliphilus* spp. (Fig. 4D).

**Figure 5:**
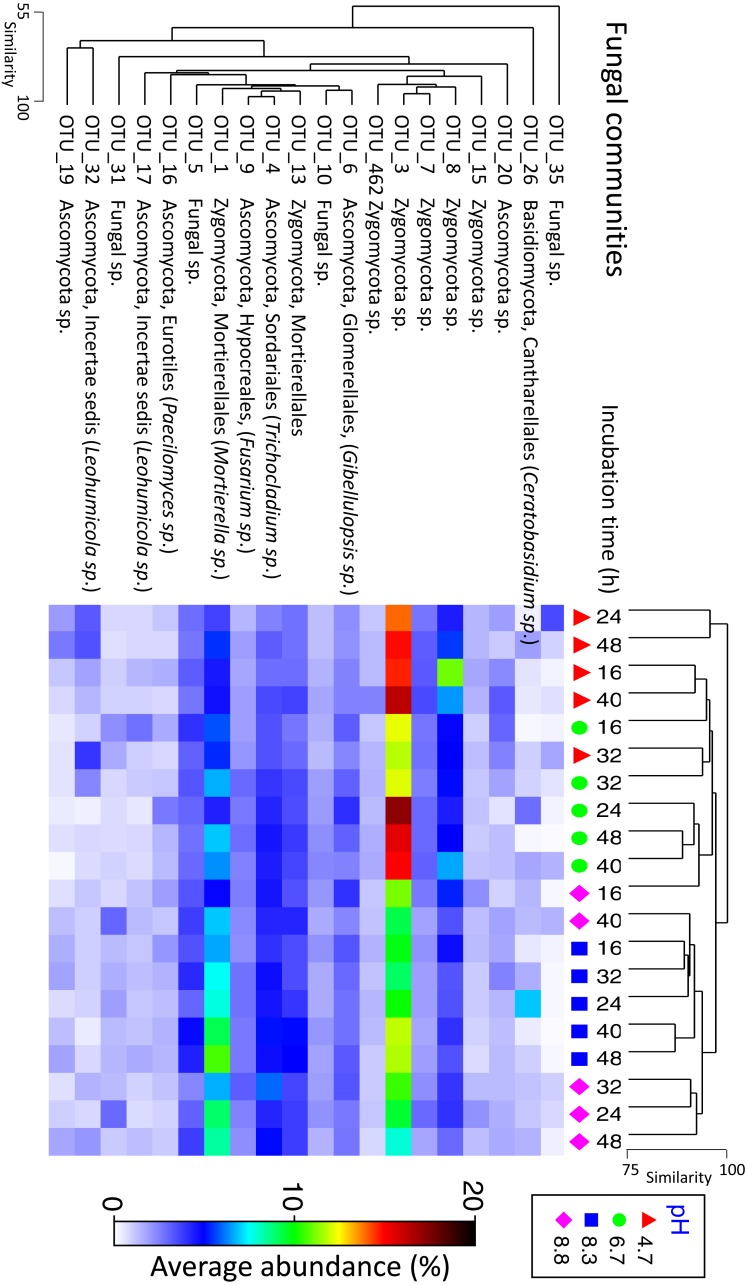
Matrix plot representing the relative abundance and clustering of the 20 fungal OTUs with the highest contribution to total sequence counts across incubation time and pH treatment. Samples have been clustered via Bray Curtis similarity measures representing the entire set of OTUs, while the 20 OTUs presented have been clustered via an index of association. Average ‘abundance’ should be treated as a relative indication only. These ‘abundances’ represent data that is non-transformed and standardised-by-total.

Fungal communities shared >70% similarity across all samples regardless of pH and time (Fig. 3). Based on percentages alone, no discernible patterns could be observed for the fungal dataset (Fig. S4), however, some OTUs were displaced depending on pH. For example, Zygomycota OTU_3 was dominant in the control and pH 6.7 treatments but was partially supplanted by another Zygomycota OTU from the order Mortierellales (*Mortierella sp.*) (Fig. 5).

An identical analysis of rarefied bacterial and fungal OTUs datasets is presented in Figs. S5 to S10. A range of diversity measures have been presented in Figs. S11 and S12. An nMDS plot from a preliminary experiment showing the relationship between bacterial communities in the initial untreated (aerobic) field-moist soil and the soil microcosms incubated under anaerobic conditions with various pH treatments is presented in Fig. S13.

### Isolation of bacteria and N-use characterisation

On TSB-nitrate medium, plates were quickly dominated by fast growing colonies sharing morphologies characteristic of motile or swarming bacteria. A total of 33 isolates were screened for nitrate utilisation. Seven showed minimal growth and respiration in liquid culture, 18 showed near complete utilisation of NO${}_{3}^{-}$ accompanied by production of both NH${}_{4}^{+}$ and N_2_O, and five showed moderate utilisation of NO${}_{3}^{-}$ with low production of NH${}_{4}^{+}$ and N_2_O. One isolate showed production of NH${}_{4}^{+}$ with little or no use of NO${}_{3}^{-}$ or production of N_2_O and two isolates exhibited respiration but did not appear to utilise N. Of the 33 isolates, 22 were selected for identification by ribosomal 16S gene DNA sequencing. All were from the Firmicutes phylum, of which three were *Paenibacillus spp.* and one was a *Brevibacillus sp.* (all producing less NH${}_{4}^{+}$), while the remainder were *Bacillus spp.* (Table  ST2A).

From 1/10 diluted TSB-nitrate medium, 44 isolates were screened for nitrate utilisation. Of these, 24 showed no or minimal growth in liquid culture over 48 h incubation while 13 reduced NO${}_{3}^{-}$ to close to zero with four of these generating significant amounts of NH${}_{4}^{+}$. Another three reduced NO${}_{3}^{-}$, but to a lesser extent, while four isolates respired but did not appear to utilise N. Seventeen isolates were identified by 16S gene sequencing. Six belonged to the *Bacillus* genus, three to *Achromobacter*, six to *Acidovorax*, one to *Bosea*, and one to *Rhodanobacter* (Table ST2B).

All isolates except one of the *Bacillus sp.* had high NO${}_{3}^{-}$ utilisation compared to uninoculated controls. The *Bacillus spp.* produced up to 536 mg NH${}_{4}^{+}$ L^−1^, while the *Acidovorax spp.* produced <45 mg NH${}_{4}^{+}$ L^−1^. One *Bacillus sp.* and two *Acidovorax sp.* produced <100 mg N_2_O L^−1^, while the remaining *Acidovorax* sp. had the highest N_2_O production at 2,840 mg L^−1^ . In general, isolates that exhibited high use of NO${}_{3}^{-}$ coupled with production of NH${}_{4}^{+}$ and N_2_O exhibited an average of ∼2.5-fold higher CO_2_ production (respiration) compared with isolates that exhibited high NO${}_{3}^{-}$ use with little or no NH_4_ and N_2_O production. Relevant 16S sequences were submitted to NCBI with accession numbers assigned between MH211426 and MH211463, submission number SUB3915485 (Table S2). Six organisms were selected for future genome sequencing, three *Bacillus spp*. and three *Acidovorax spp*. (Table ST2C).

## Discussion

### The effects of rapid pH change on soil chemistry and microbiology

KOH additions and resulting pH elevation caused the concentration of DOM in soil microcosms to greatly increase (150-fold) compared to the controls. Added hydroxyl ions displaced negatively-charged organic molecules into solution. Previous work has shown that monovalent cations like K^+^ (KOH) and NH${}_{4}^{+}$ (NH_4_OH—product of urea hydrolysis) are much more effective in solubilising organic matter than divalent cations such as Ca^2+^ (Ca(OH)_2_) (Curtin, Peterson & Anderson, 2016). The amount of DOM released at elevated pH in these experiments was concordant with our previous research using the same methodology (Anderson, Peterson & Curtin, 2017), as were the higher respiration rates.

Over medium to long time scales (months to years), pH is known to be a dominant environmental variable that shapes soil microbial communities (Lauber et al., 2009; Zhalnina et al., 2015). Changes in pH are also known to cause shifts in active organisms over short timescales (Brenzinger, Dörsch & Braker, 2015). Although the strongest predictor for both bacterial and fungal community change in these experiments was pH, community change may also be indirectly influenced by the effect that pH has on DOM release. Theoretically, high levels of DOM released via increased pH should benefit copiotrophs (*r*) over oligotrophs (*K*) (Fierer, Bradford & Jackson, 2007; Goldfarb et al., 2011), but at the same time elevated pH is likely to alter cellular homeostasis, regulation of nutrient availability, or other factors such as salinity, metal accessibility, or organic C characteristics (Lauber et al., 2009).

Previous analysis of the soil used in this study suggests that a mix of carbon sources are released as pH increases, of which 45% are bioavailable. These range from labile hexose and pentose sugars to more recalcitrant polyphenolic molecules (Curtin, Peterson & Anderson, 2016). The lack of proportionality between respiration rates and DOM released in this study suggests that higher amounts of bioavailable C did not lead to higher biomass, instead the microbial community and associated metabolic response has shifted toward more copiotrophic organisms. Addition of low molecular weight C compounds (glucose, citric acid, glycine) to soil has been previously observed to shift the structure of bacterial communities to more copiotrophic organisms (Eilers et al., 2010) with no strong correlations between respiration rates and community structure. Community changes and catabolic responses may be unlinked because some C-substrates are preferentially used without biomass changes (Devevre & Horwath, 2000).

Proteobacteria are abundant in high C soils (Fierer, Bradford & Jackson, 2007) with β and γ − Proteobacteria considered important soil copiotrophs (Eilers et al., 2010) in conjunction with Firmicutes and Actinobacteria (Zhalnina et al., 2015). Bacteroidetes and β-Proteobacteria are initial metabolisers of labile soil-C (Padmanabhan et al., 2003) and increases in the abundance of these organisms have been correlated with C mineralisation rates (Fierer, Bradford & Jackson, 2007). Our study is consistent with regard to expansion of Bacteriodetes (*Flavobacteriales*), specifically in the control and pH 6.7 microcosms (∼5.5-fold-increase in DOM with ∼2-fold increase in respired CO_2_), suggesting that the microbial community does respond to higher concentrations of bioavailable C at pH values <7.

In general, we observed a decrease in α-Proteobacteria (specifically *Rhizobiales*), Actinobacteria and Acidobacteria OTUs at all pH and DOM values, while a few β- and γ-Proteobacteria OTUs increased. Our results suggest that the chemical changes induced by KOH addition to soil are comparable to soils where pH and DOM are elevated due to higher urea inputs. Niche differentiation occurs in soil where higher bovine density (and presumably urea inputs) induces increases in pH and total organic carbon, with Actinobacteria, α-Proteobacteria and Verrucomicrobia decreasing and Bacteriodetes increasing (Philippot et al., 2010; Philippot et al., 2009a; Philippot et al., 2009b).

In the study by Fierer, Bradford & Jackson (2007), abundance of Firmicutes could not be predicted by C-mineralisation (nor other measured soil parameters), while in the Park Grass experiment in the UK, total C and N and pH were negatively correlated with Firmicutes (Zhalnina et al., 2015). Our study differs from the literature with regard to the Firmicutes as they are the most responsive to pH and DOM increases. Our experimental conditions are quite different with the combined complexity of alkaline pH and anaerobic conditions likely playing a larger role than just DOM in defining niche differentiation and shaping microbial community structure (Banerjee et al., 2016; Husson, 2013; Pett-Ridge & Firestone, 2005).

Comparisons of OTU distributions in our study indicate that the dominant feature driving sample dissimilarities was large increases in Firmicutes from the classes Bacilli and Clostridia. Large expansion of Firmicutes, first dominated by *Bacillales* (up to 46%) and then followed by *Clostridiales* (up to 53%), (along with large decreases in Proteobacteria) have been observed in alkaline soil crusts (pH 8.5) that were rehydrated and incubated under dark anoxic conditions (Angel & Conrad, 2013). Our results are also concordant with O’Callaghan et al. (2010), who observed a 38% increase in Firmicutes, and decreases in Proteobacteria (18%), Acidobacteria (8%), Actinobacteria (5%), and Bacteriodetes (5%), in soil where pH rose to values of >8, two days after bovine urine addition.

Genus level identifications from the Bacilli and Clostridia in our study included *Bacillus*, *Paenibacillus, Tepidibacter*, *Alkaliphilus and Clostridium*. Cultured examples of these organisms from the literature include facultative anaerobes (and obligate anaerobes) that are highly responsive to more recalcitrant C-sources, are either alkaliphilic or alkalotolerant, and show fermentative type metabolisms (Chen, Song & Dong, 2006; Goldfarb et al., 2011; Lee et al., 2007; Slobodkin et al., 2003; Urios et al., 2004). *Bacillus* species and related genera can be found in a wide variety of habitats. *Bacillus* and *Paenibacillus* species can be considered as drivers of soil organic matter mineralisation, are frequently abundant in situations where C and N are not limited and are capable of degrading polymeric carbonaceous substances (Mandic-Mulec, Stefanic & Van Elsas, 2015). Although there is some evidence to suggest that *Clostridium* are generally acid loving (Kuhner et al., 2000), the related clostridial OTUs (*Tepidibacter* and *Alkaliphilus*) identified in this study suggest a wider range of pH tolerance (Lee et al., 2007; Slobodkin et al., 2003; Urios et al., 2004).

Relative changes in fungal populations in response to pH change were smaller than for bacteria, with ∼28% of fungal variation attributed to pH, versus ∼57% for bacteria. Only ∼6.6 percent of the variation in fungal communities could be attributed to incubation time. Fungal communities are known to be less responsive to pH than bacteria (Lauber et al., 2009; Rousk et al., 2010a) and fungal abundance has been found to be negatively correlated with pH (Rousk, Brookes & Baath, 2010b), but positively correlated with C and N additions (Banerjee et al., 2016). For example, investigations of the response of fungal (and bacterial) communities to ovine urine (where pH increased from ∼3.5 up to 6.5 and DOC increased from ∼0 up to ∼2,000 mg kg^−^1) indicates no fungal biomass change (Nunan et al., 2006; Williams, Grayston & Reid, 2000), no significant correlation between biomass and pH, NH${}_{4}^{+}$ or NO${}_{3}^{-}$, but weak correlation between biomass and DOC (Singh, Nunan & Millard, 2009). In pH 6.1 soils, Banerjee et al. (2016) noted that although fungal biomass increased, the richness, evenness, and diversity decreased within 4 days after organic matter and nutrient addition leading to ‘keystone’ fungal species being favoured. Singh, Nunan & Millard (2009) suggest that because fungi are capable of degrading complex organic carbon they are less responsive to short term changes in nutritional availability.

### Soil N processes and their relationship to microbiology following pH change

Although the possibility exists that nitrifier-denitrification contributed to N_2_O production during the incubation period in the control and pH 6.7 microcosms, the likelihood is low given the elevated DOM concentrations, the saturated conditions and the low concentrations of native NH${}_{4}^{+}$ available for oxidation (Wrage et al., 2001). Under saturated conditions (WFPS 90%), experiments by Kool et al. (2011) indicated that >92% of total N_2_O is derived from ‘conventional’ denitrification of NO${}_{3}^{-}$ and our results are broadly concordant given that NO${}_{3}^{-}$ was consumed and N_2_O was produced. There were however variations in the amount of time required for the original supply of native NO${}_{3}^{-}$ to be consumed. Native NO${}_{3}^{-}$ was nearly completely utilized within the first 16 to 24 h in the control, pH 6.7 and pH 8.3 microcosms, but in the pH 8.8 microcosms, NO${}_{3}^{-}$ did not decline until after 32 h. When additional NO${}_{3}^{-}$ was added to measure denitrification rates, maximum DEA occurred in microcosms that had been incubated for at least 24 h. The DEA assay measures only N_2_O produced during the assay period as the anaerobic headspace was refreshed. For all treatments, DEA potential declined at incubation times greater than 24 h. This may have been due to extended periods (i.e., >24 h) of low NO${}_{3}^{-}$ concentrations prior to the DEA assays leading to a decline in NO${}_{3}^{-}$ linked translation of denitrification genes such as N_2_O-R and nitrate reductase (Moreno-Vivian et al., 1999; Zumft, 1997). Alternatively, the microbial communities that developed with increasing incubation times may have expressed different denitrification phenotypes (Dorsch, Braker & Bakken, 2012; Sanford et al., 2012).

It also seemed that the relative metabolic contribution of denitrification declined over time as NO_3_^−^ was utilized, giving the opportunity for other anaerobic metabolisms such as fermentation to have proportionally greater influence. For example, in the pH 8.3 treatment, the respiration rate in microcosms incubated for 24 h was 40% lower than those incubated for 48 h, yet at 24 h denitrification rates were ∼4.5-fold higher. *Bacillus* OTUs dominated at 24 h where the lower respiration rates and higher denitrification was observed, but *Bacillus* was then displaced by a consortium of clostridial species after 48 h. Given that *Clostridia* can be obligate or facultative anaerobes, we think that this species displacement is a response to the changing chemical conditions in the microcosms marked by nitrate depletion, elevated pH and sustained anaerobicity.

In contrast to the KOH amended microcosms, the N_2_O/(N_2_O + N_2_) ratio in the control microcosms equalled 1, indicating that this treatment did not have active N_2_O-R. Liu et al. (2010a) and Bakken et al. (2012a) have previously shown that production of functional N_2_O-R depends on the post-transcriptional pH being greater than 6.1, which is consistent with our results. In an agricultural environment this raises interesting ecological questions, because urea hydrolysis happens to elevate pH for several days which would immediately alleviate any post-transcriptional interference of *nos* Z expression and allow rapid production of functional N_2_O-R.

We observe full denitrification of NO${}_{3}^{-}$ to N_2_ within 16 h and maximum rates after 24 h which indicates that suitable redox conditions for denitrification were established quickly in our microcosms and a corresponding rapid genetic and enzymatic response followed. The predominant electron acceptors in a weakly reducing environment are O_2_, NO${}_{3}^{-}$ and manganese oxide (MnO_2_) (Uteau et al., 2015) with the threshold between oxic and anoxic soil lying somewhere between 300 and 400 mV. These conditions develop in response to high soil moisture contents that slow down gas diffusion (e.g., post irrigation or flooding) and there are good correlations between N_2_O flux and relative soil gas diffusivity (D_p_/D_O_) (Hansen, Clough & Elberling, 2014; Owens et al., 2017; Owens et al., 2016). Biologically, low O_2_ concentrations, or restricted diffusion of oxygen would trigger rapid induction of *de novo* denitrification enzyme synthesis depending on pH. *De novo* enzyme synthesis follows a sequential order, with nitrate reductase formed within 2–3 h, nitrite reductase between 4–12 h and N_2_O-R between 24 and 42 h (Dendooven & Anderson, 1994; Dendooven & Anderson, 1995; Firestone & Tiedje, 1979; Smith & Tiedje, 1979). Recent investigations have observed even earlier synthesis of N_2_O-R than 24–42 h, with peaks in gene transcripts for *nos* Z (and presumably translation of N_2_O-R) occurring within <10 h (Liu, Frostegard & Bakken, 2014).

The denitrification trait is spread over a wide taxonomic range including bacteria, archaea and some eukaryotes (Zumft, 1997). We observed large proliferations of Firmicutes in conjunction with peaks in DEA. Denitrification and/or reduction of nitrate/nitrite is common in cultured *Bacillus* spp. and they have been shown to be numerically important culturable members of denitrifying communities in agricultural soils (Jones et al., 2011; Verbaendert et al., 2011). The closely related *Paenibacillus* (pH 8.3 microcosms) are also capable of heterotrophic nitrification, dissimilatory NO${}_{3}^{-}$ reduction to NH${}_{4}^{+}$ (DNRA), and full denitrification and grow optimally in neutral to alkaline pH conditions (Behrendt et al., 2010). Like *Paenibacillus*, some *Bacteriodetes* (as observed in the pH 6.7 microcosms) have N_2_O-R and have been observed to fully denitrify NO${}_{3}^{-}$ to N_2_ (Horn et al., 2005). To date, culture independent studies have not shown Firmicutes to be numerically important in denitrification, however, PCR primers and lysis techniques may not be effective for these bacteria, thereby artificially reducing their relative contribution (Verbaendert et al., 2011).

### Potential for DNRA and fermentation in the microcosms

After pH change, the microcosms had high DOC/NO_3_^−^ ratios with no correlations evident between estimated DOC mineralisation and NO${}_{3}^{-}$ nor CO_2_ respiration. There was also a mismatch between increasing NH${}_{4}^{+}$ relative to consumed NO${}_{3}^{-}$, especially in the pH 8.8 microcosms, suggesting that other anaerobic metabolisms were active aside from denitrification. DNRA is an energy yielding anaerobic process that is favoured in C-replete conditions when NO${}_{3}^{-}$ becomes limiting (C/NO${}_{3}^{-}$ ratio > 12) (Giles et al., 2012; Rutting et al., 2011). Using the DNRA stoichiometry in equation [2] presented by Lam et al. (2009), if all available native NO${}_{3}^{-}$ in our microcosms (∼20 mg kg^−1^ NO${}_{3}^{-}$-N) was reduced via DNRA (i.e., ignoring NO${}_{3}^{-}$ also needed for denitrification) then approximately 26 mg kg^−1^ NH${}_{4}^{+}$ could be produced, yet we observed up to 50 mg kg^−1^. The significant amounts of additional N required to balance the N requirements in our experiments are likely to be derived from the ample supplies of DON and DOC in the microcosms that could undergo depolymerisation and ammonification (Burger & Jackson, 2004; Rutting et al., 2011; Schimel & Bennett, 2004).

*Bacillus* species are well known as nitrate reducers and N_2_O emitters, but many strains do not produce N_2_O after NO${}_{3}^{-}$/NO${}_{2}^{-}$ reduction (Verbaendert et al., 2011). DNRA is known to occur in a number of *Bacillus* species with varying concentrations of N_2_O produced (Heylen & Keltjens, 2012; Mania et al., 2014; Nakano et al., 1998; Sun, De Vos & Heylen, 2016). Although genome information is not yet available, possible N-metabolisms for the predominantly *Bacillus* species isolated from the microcosms in this study include denitrification, DNRA and possibly N_2_ fixation. These isolates produced an excess NH${}_{4}^{+}$ and N_2_O compared to uninoculated controls suggesting DNRA could be a dominant metabolism. Given the concentration of NO${}_{3}^{-}$ available in the medium, additional N is still required to support the concentrations of NH_4_^+^ and N_2_O produced.

The production of NH${}_{4}^{+}$ and N_2_O at concentrations beyond that supplied by mineral-N in both microcosms and cultures allows speculation that depolymerisation and ammonification of organic matter is active (Curtin, Peterson & Anderson, 2016), along with the possibility of co-denitrification (biological and/or chemical) where 50% of the N in N_2_O (and N_2_) is derived from labile (nucleophilic) organic N such as amines (Phillips et al., 2016; Selbie et al., 2015; Spott, Russow & Stange, 2011). Biogenic amines can also be derived through fermentation, with *Bacillus spp.* being common producers (Chang & Chang, 2012).

The other main driver of treatment differences were organisms from the order Clostridiales.  Tiedje (1988) describes obligate anaerobic DNRA capable *Clostridium* spp. (Caskey & Tiedje, 1980; Keith, Macfarlane & Herbert, 1982). Clostridia are also well known for their fermentative metabolisms that have been exploited for over 100 years (Moon et al., 2016; Wiegel, Tanner & Rainey, 2006), which combined with the observations that there was no correlation between NO${}_{3}^{-}$ consumption and CO_2_ production adds support to the theory that metabolisms aside from denitrification/DNRA operate in these microcosms, especially beyond 40 h when Clostridiales start displacing Bacilliales. Fermentation is also known to occur in *Bacillus* species, specifically the well-studied *B. subtilis* (Ramos et al., 2000).

Recent research has shown that fermentative organisms (Clostridiales) influence the competition between denitrifiers and DNRA bacteria through competition for fermentative C-substrates (electron donors). Higher ratios between substrates and nitrate leads to a combination of fermentation and DNRA (both fermentative and respiratory) with no denitrification. When the ratio between substrates and nitrate lowers, denitrification takes a larger role until it eventually out-competes both fermentation and DNRA (Van den Berg et al., 2017a; Van den Berg et al., 2017b). Comparing these studies to our microcosms is problematic as the C-sources in our study are so diverse. However, the biogeochemical evidence indicates that NO${}_{3}^{-}$ quickly declines while DOM remains high which would lead to a higher substrate/nitrate ratio and thus DNRA and fermentation taking a dominant role. This possibility is further supported by increases in NH${}_{4}^{+}$ and CO_2_ respiration rates beyond the peaks in DEA. There is also the possibility that DNRA has a more significant role that we envisage and that DEA measurements reflect reduction of N_2_O via ‘atypical nosZ’ (Giblin et al., 2013; Jones et al., 2013; Jones et al., 2011; Samad et al., 2016b; Sanford et al., 2012).

### Implications for urea impacted soil

The pH changes induced by our KOH additions in this study are representative of what could be expected in the field under animal urine patches or in the vicinity of urea fertilizer prills (Clough et al., 2010; O’Callaghan et al., 2010). Increases in electrical conductivity (EC) associated with elevated pH were high at 6 mS cm^−1^, but were not out of the ordinary when compared to other studies investigating urine additions to soil, nor is the associated release of excess SOM (Curtin, Peterson & Anderson, 2016; Haynes & Williams, 1992). Recent research documenting N_2_O emissions in urea-amended saturated soils with elevated pH and declines in O_2_ and redox changes, also suggest that this work has direct relevance to what would be expected under field conditions (Hansen, Clough & Elberling, 2014; Owens et al., 2017; Owens et al., 2016).

Given that there is experimental and field evidence for chemical conditions conducive to denitrification in the days following urea application, this supports the idea that N-cycling in soils ideally should not be considered a sequential process, but instead is highly dynamic, with N-processing dependent on resources available and the physicochemical environment. Our work suggests that N-resources can be quickly supplied from both organic and inorganic sources with the distinct possibility that significant N could be lost as N_2_ shortly after urea deposition. It is unknown what happens to the large excess of SOM released at high pH that is not metabolised during these short-term incubations. We should be aware that pulses of fresh C into soil can lead to loss of native C (priming) with some research indicating that excess N-fertilisation (and possibly intensification) leads to soil C declines (Kuzyakov, Friedel & Stahr, 2000; Mau et al., 2015).

Although pH is a universal mechanism that selects for microbial communities, the response to pH will vary according to soil types. The microbial phenotypes expressed will also be dependent on the available soil C and N resources with multiple N-transformation pathways possible. For this particular soil, pH elevation and anoxia allowed Firmicutes bacteria to flourish and contribute to rapid processing of N resources. Investigations of the ratios between various N-metabolisms in these microcosms would require isotope labelling with more defined experiments required to understand short and long-term cascades of biological N-processing and the transient ecologies driving N-transformations as soil conditions stabilise post urea addition.

## Conclusions

At soil pH values representing expected deviations induced by either urine or urea prills, we observed large increases in DOM, respiration and DEA potential within <24 h of pH change. DEA potential was such that in order to balance the concentrations of NH${}_{4}^{+}$ and N_2_O concentrations produced there is a requirement for mineralisation of DOM to supplement the available NO${}_{3}^{-}$ resources. The microbial community structure changed dramatically in response to the new soil chemical regime, specifically moving towards a dominance of *Firmicutes* bacteria. The large increase in *Firmicutes* bacteria coincided with the highest DEA potential, while the cultured representatives of *Firmicutes* bacteria had inferred metabolisms that including denitrification and dissimilatory nitrate reduction to ammonia (DNRA).

##  Supplemental Information

10.7717/peerj.6090/supp-1 Supplemental Information 1 Supplementary information This is a supplemental file containing pertinent figures and tables organized in ascending numerical order as cited in the text of the main publication. Each figure or table in this file contains explanatory information describing what each represents.

10.7717/peerj.6090/supp-2 Supplemental Information 2 Raw chemical data This file contains raw data underpinning Table 1, Fig. 1 and Fig. S2

10.7717/peerj.6090/supp-3 Supplemental Information 3 Raw data included in Table 1, Fig. 1 and Fig. S1

10.7717/peerj.6090/supp-4 Supplemental Information 4 Raw chemical data used for assessing pre-DEA incubation times This file contains data used to determine optimal incubation time periods to investigate DEA that allowed for expression of denitrification gene activity (biogeochemical detection of activity via N_2_O and N_2_ production).

10.7717/peerj.6090/supp-5 Supplemental Information 5 CO_2_ measurements also accounting for that dissolved as carbonates This file contains raw data of both CO_2_ measured and that liberated from carbonates via acidification.

10.7717/peerj.6090/supp-6 Supplemental Information 6 Summary of chemical data This file contains data from experiments 3 and 4 summarized into one file with averages and standard deviations calculated and exploratory graphs.

10.7717/peerj.6090/supp-7 Supplemental Information 7 OTU Input file for PRIMER 7 statistical package Data represents raw sequence counts which were then standardised by total in PRIMER 7 prior to analysis.

10.7717/peerj.6090/supp-8 Supplemental Information 8 OTU identification table This data represents the OTU information with Megablast ID.

## Additional Information and Declarations

### DNA Deposition

The following information was supplied regarding the deposition of DNA sequences: Relevant 16S sequences are available at GenBank, accession numbers assigned between MH211426 and MH211463.

### Data Availability

The following information was supplied regarding data availability: The raw data are available as Supplemental Files.
