# Supplementary material for: Rapid increases in soil pH solubilise organic matter, dramatically increase denitrification potential and strongly stimulate microorganisms from the Firmicutes phylum
Source: PeerJ. 2018 Dec 12;6:e6090. doi: 10.7717/peerj.6090 (PMC6295159; doi:10.7717/peerj.6090)
Supplement: Supplemental Information 1 — This is a supplemental file containing pertinent figures and tables organized in ascending numerical order as cited in the text of the main publication. Each figure or table in this file contains explanatory information describing what each represents. [file peerj-06-6090-s001.pdf]

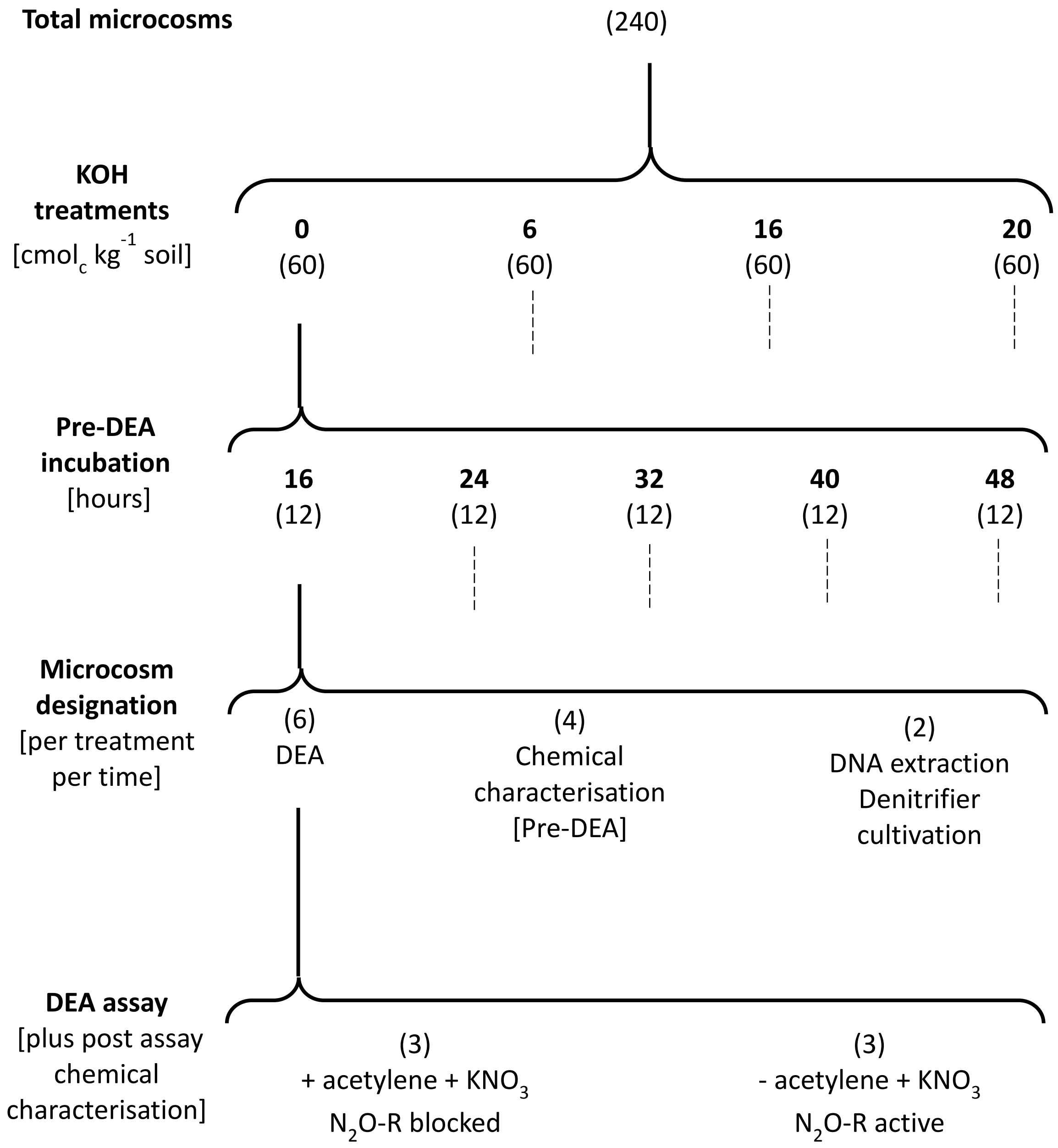

Supplementary Figure S1: Microcosm treatment structure. There was a total of 240 microcosms, 60 for each of the four KOH treatment, giving 12 microcosms for each of the five incubation times. Microcosms were incubated anaerobically and destructively sampled. At each time point, 4 microcosms were used for chemical characterisation, and 2 were used as inoculums for isolation of denitrifying microorganisms and DNA extraction to determine microbial community structure. The remaining 6 microcosms at each time point were used for the denitrification enzyme activity (DEA) assay, 3 with acetylene added to block nitrous oxide reductase activity and 3 without to gauge the activity of the nitrous oxide reductase.

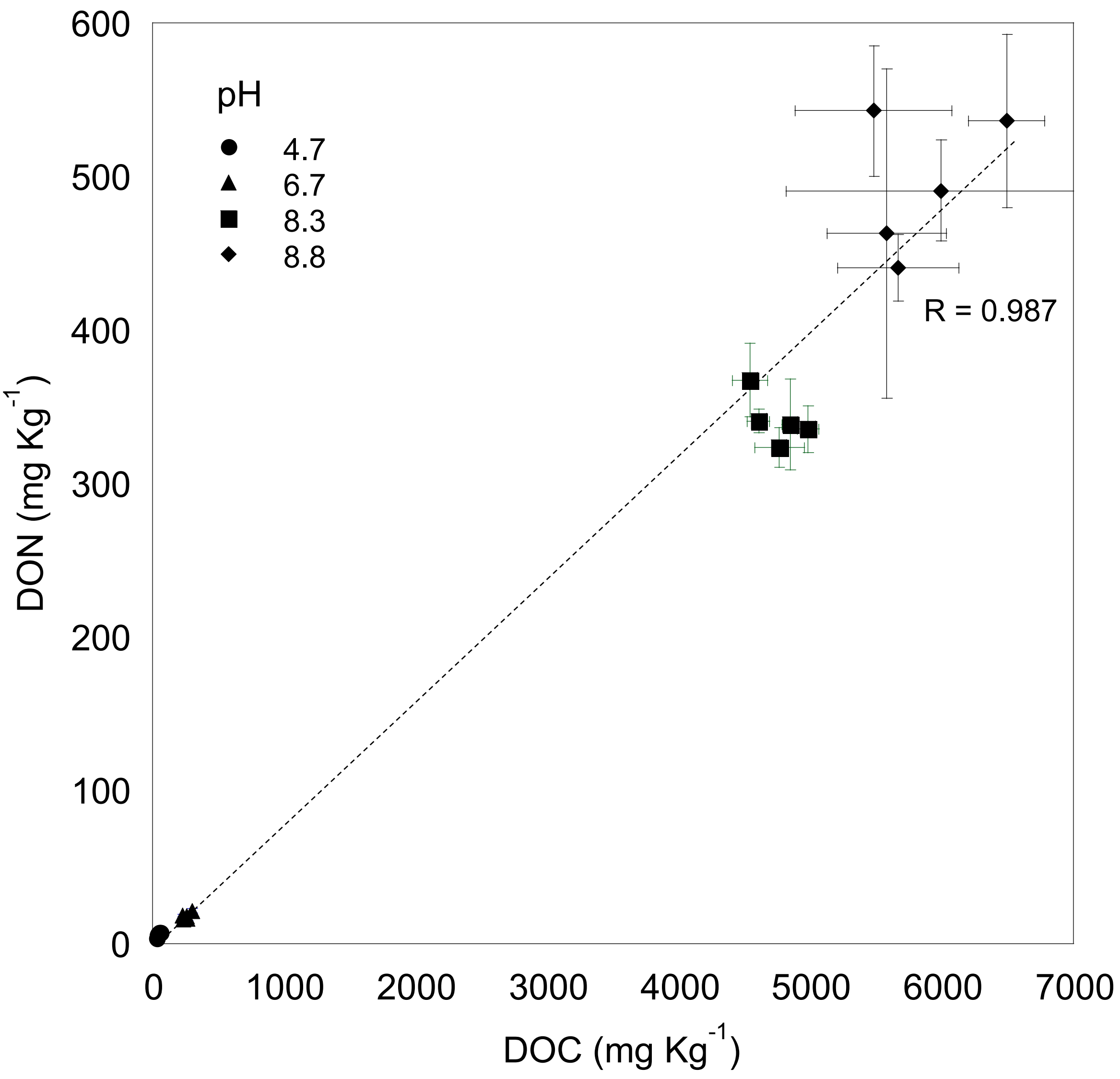

Supplementary Figure S2: DOC and DON correlation when KOH is used to alter soil pH.

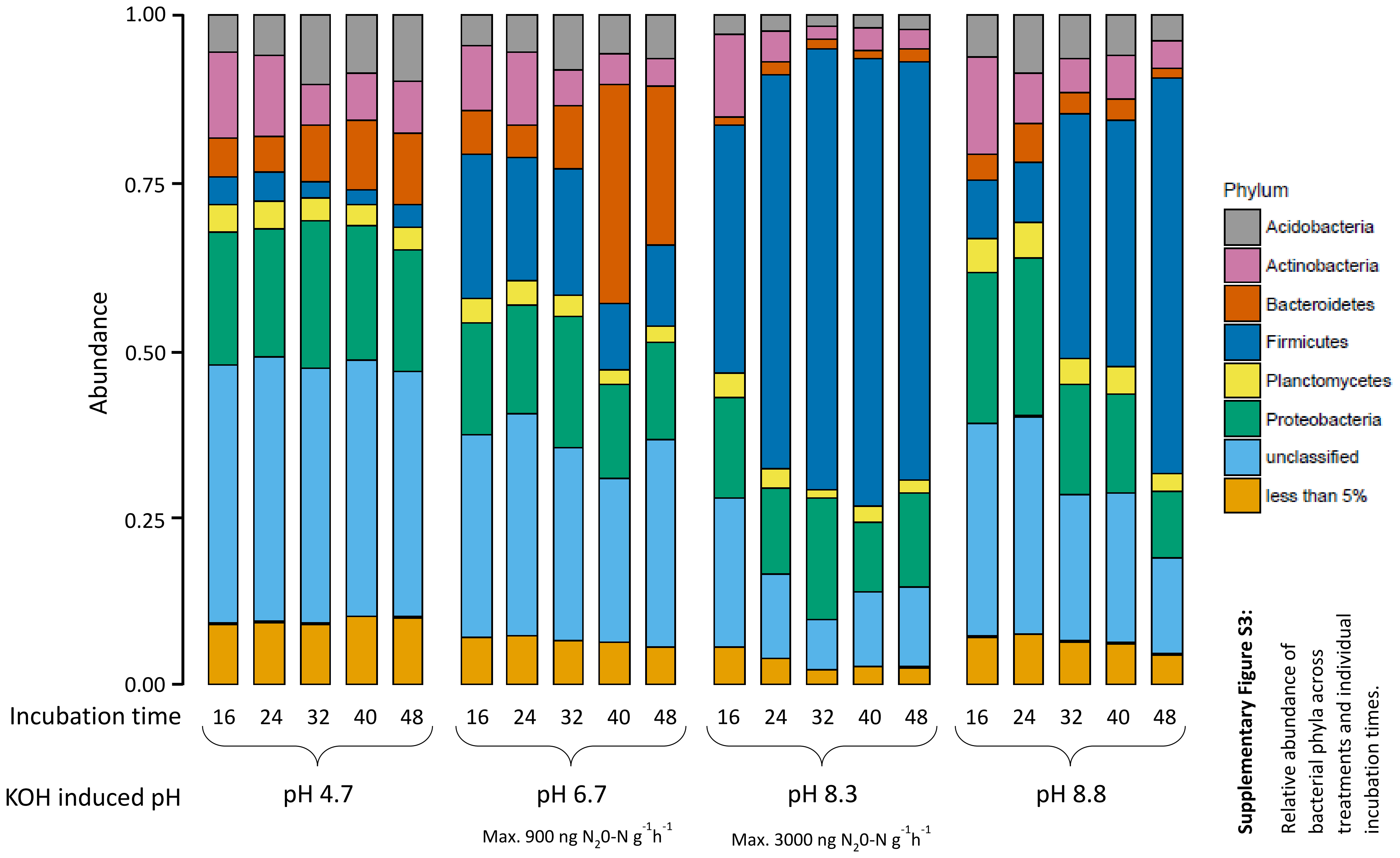

Supplementary Figure S3:

Relative abundance of bacterial phyla across treatments and individual incubation times.

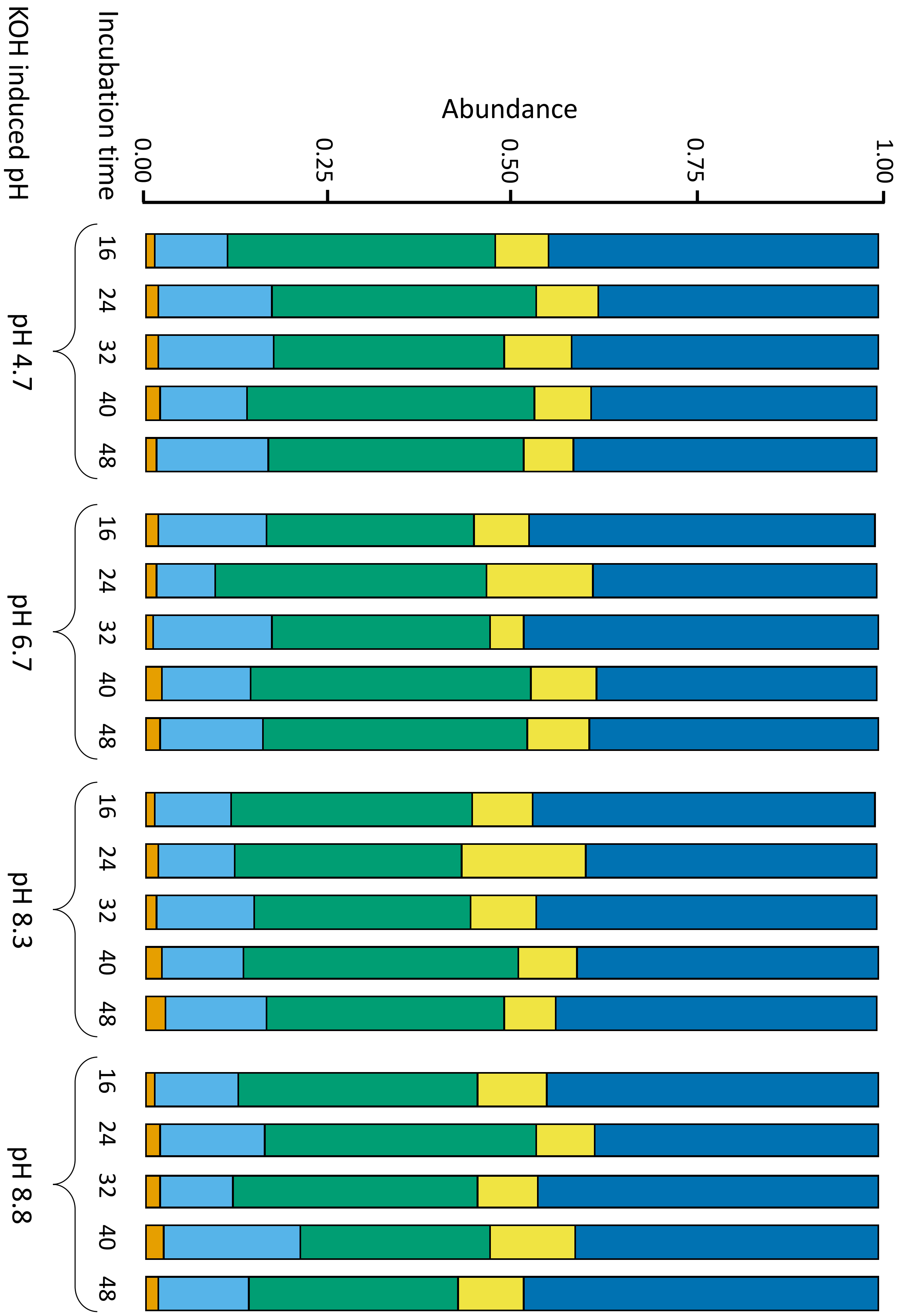

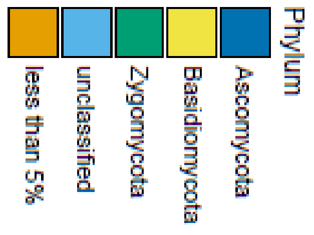

Supplementary Figure S4:

Relative abundance of fungal phyla across treatments and individual incubation times.

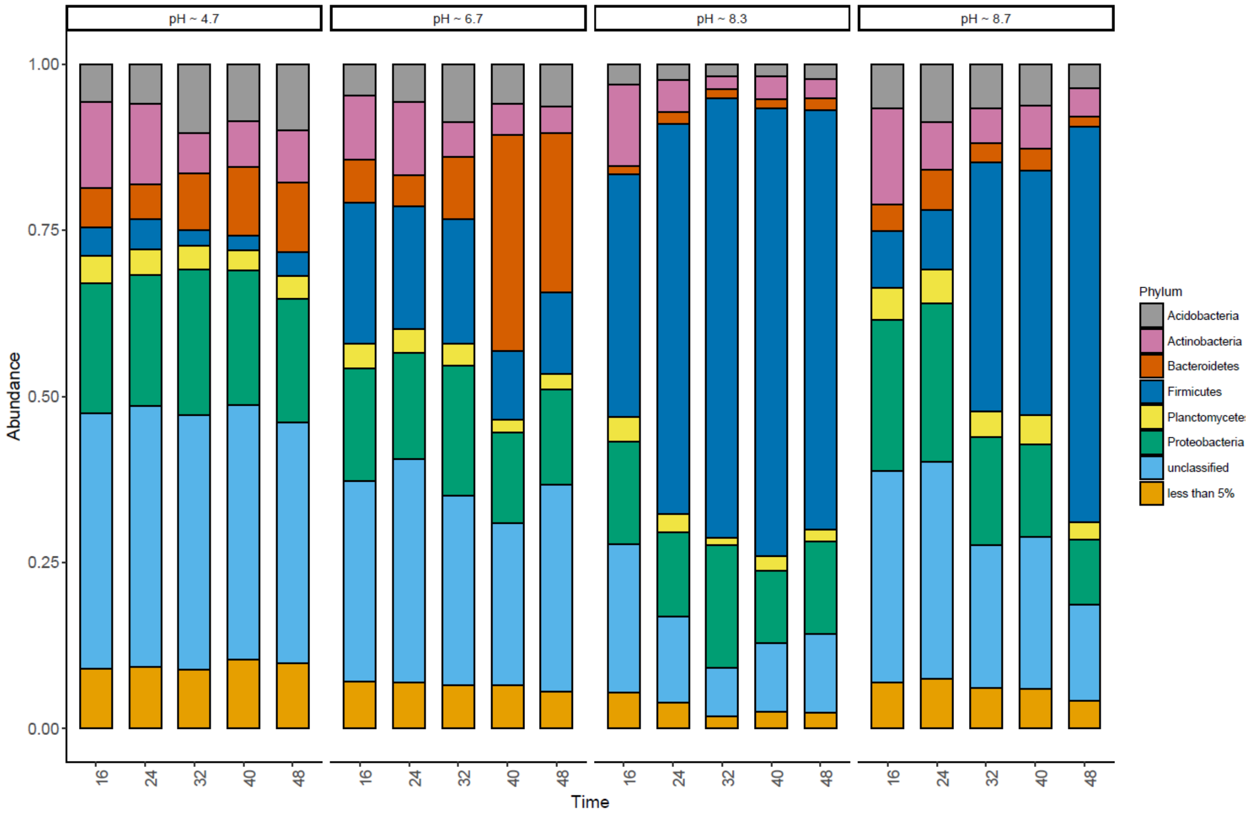

Supplementary Figure S5: Relative abundance of bacterial phyla across treatments and incubation time after dataset was rarefied. Note that there is little difference when compared to the full dataset normalised by proportion.

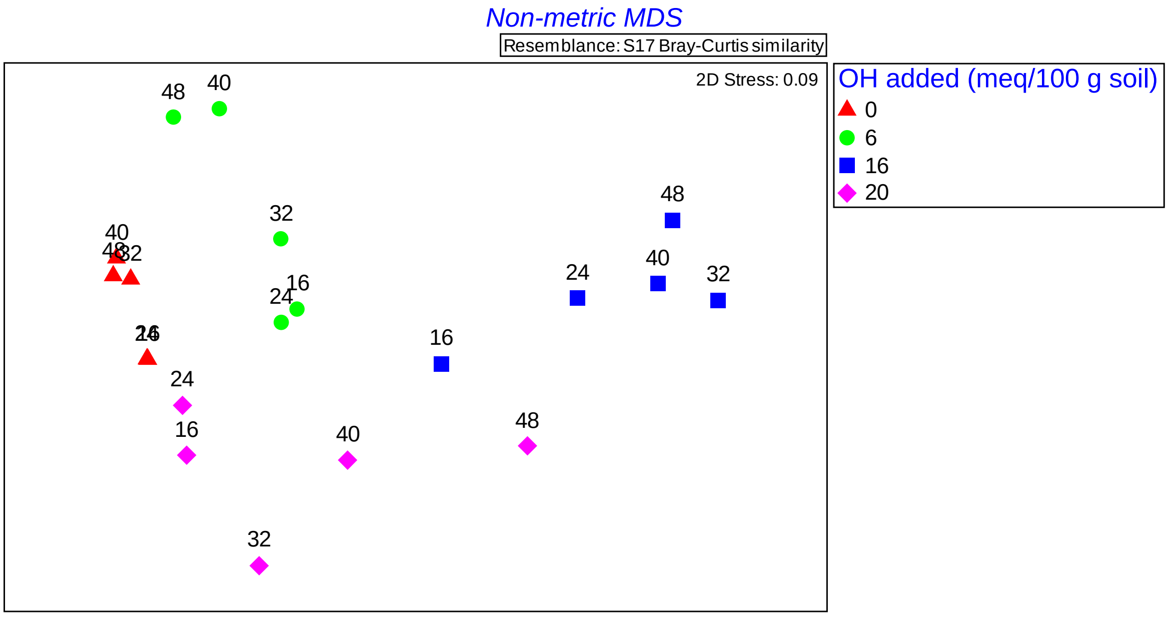

Supplementary Figure S6: MDS plot of bacterial communities after dataset was rarefied. Note that there is little difference when compared to the full dataset normalised by proportion. 0, 6, 16, and 20 “OH added” represents pH 4.7, 6.7, 8.3 and 8.8 respectively.

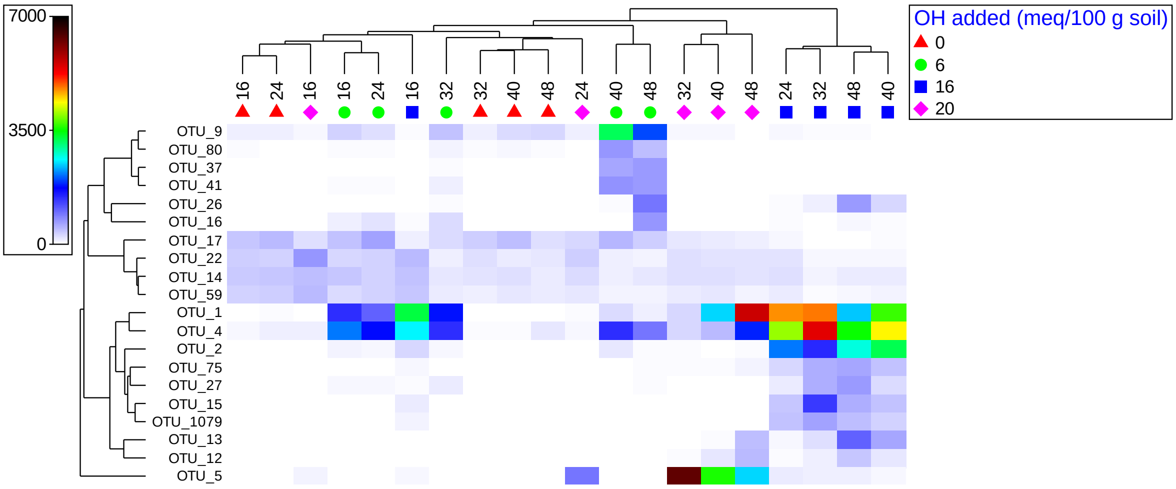

Supplementary Figure S7: Combined heat-map of changes in the most abundant bacterial OTUs across all treatments and incubation times. Note that there is little difference when compared to the full dataset normalised by proportion – the same OTUs are represented. 0, 6, 16, and 20 "OH added" represents pH 4.7, 6.7, 8.3 and 8.8 respectively.

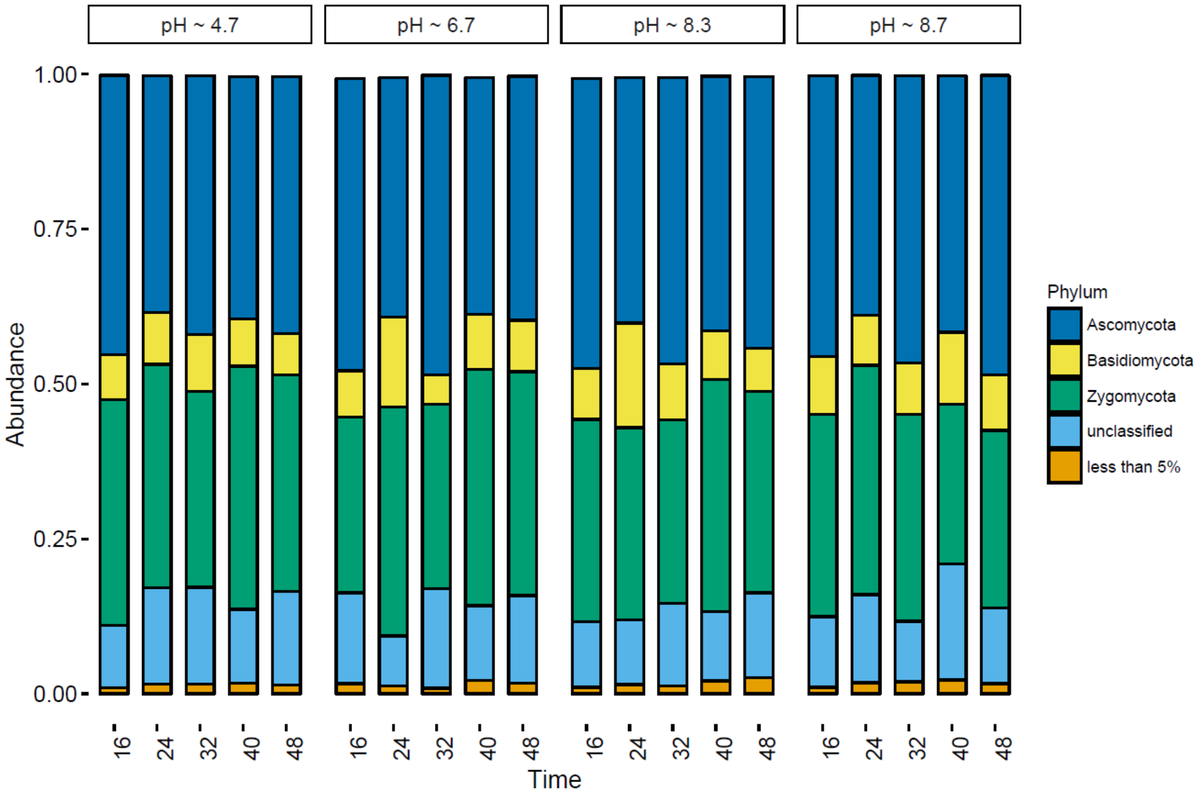

Supplementary Figure S8: Relative abundance of fungal phyla across treatments and incubation time after dataset was rarefied. Note that there is little difference when compared to the full dataset normalised by proportion.

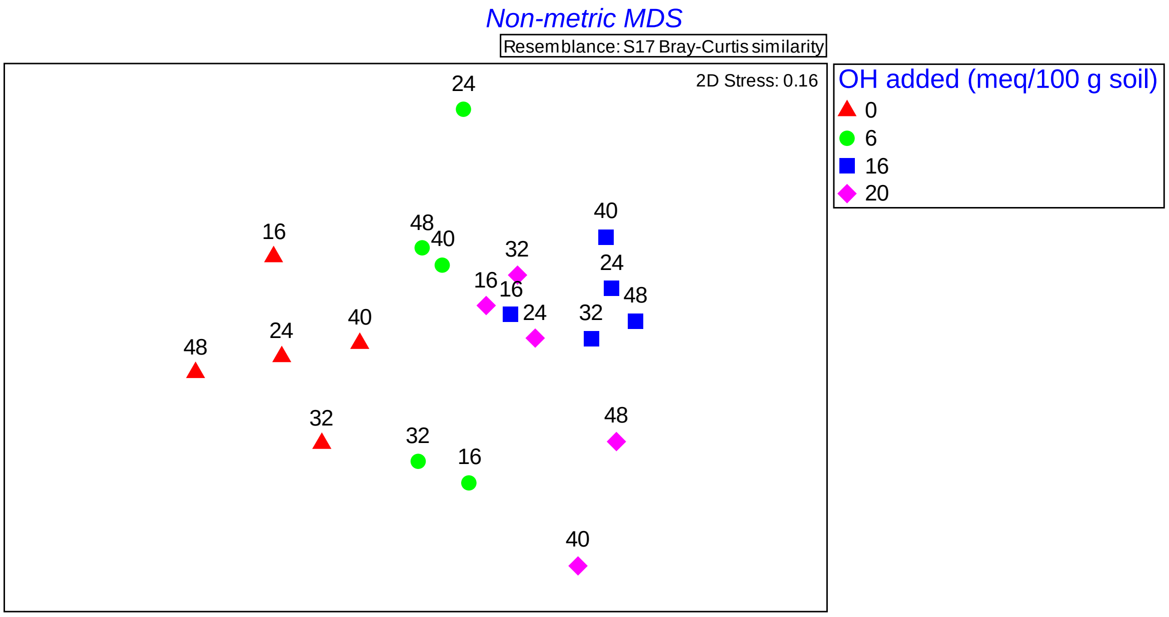

Supplementary Figure S9: MDS plot of fungal communities after dataset was rarefied. Note that there is little difference when compared to the full dataset normalised by proportion. 0, 6, 16, and 20 "OH added" represents pH 4.7, 6.7, 8.3 and 8.8 respectively.

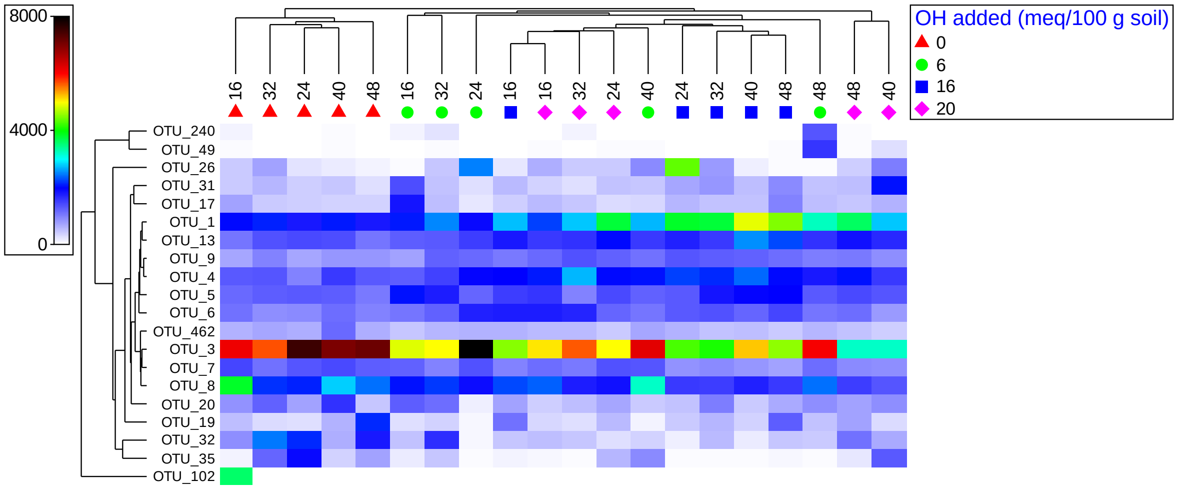

Supplementary Figure S10: Combined heat-map of changes in the most abundant fungal OTUs across all treatments and incubation times. Note that there is little difference when compared to the full dataset normalised by proportion - the same OTUs are represented. 0, 6, 16, and 20 "OH added" represents pH 4.7, 6.7, 8.3 and 8.8 respectively.

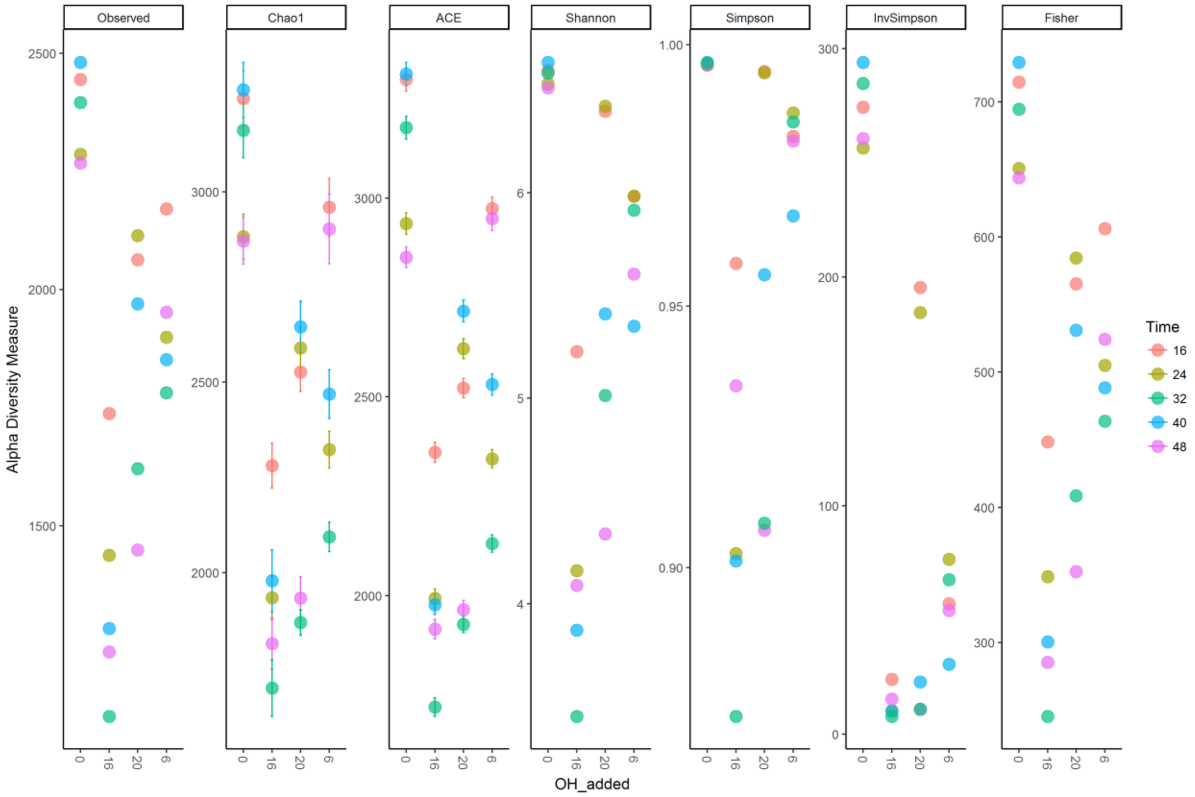

Supplementary Figure S11: Diversity measures for bacterial OTUs. 0, 6, 16, and 20 "OH added" represents pH 4.7, 6.7, 8.3 and 8.8 respectively.

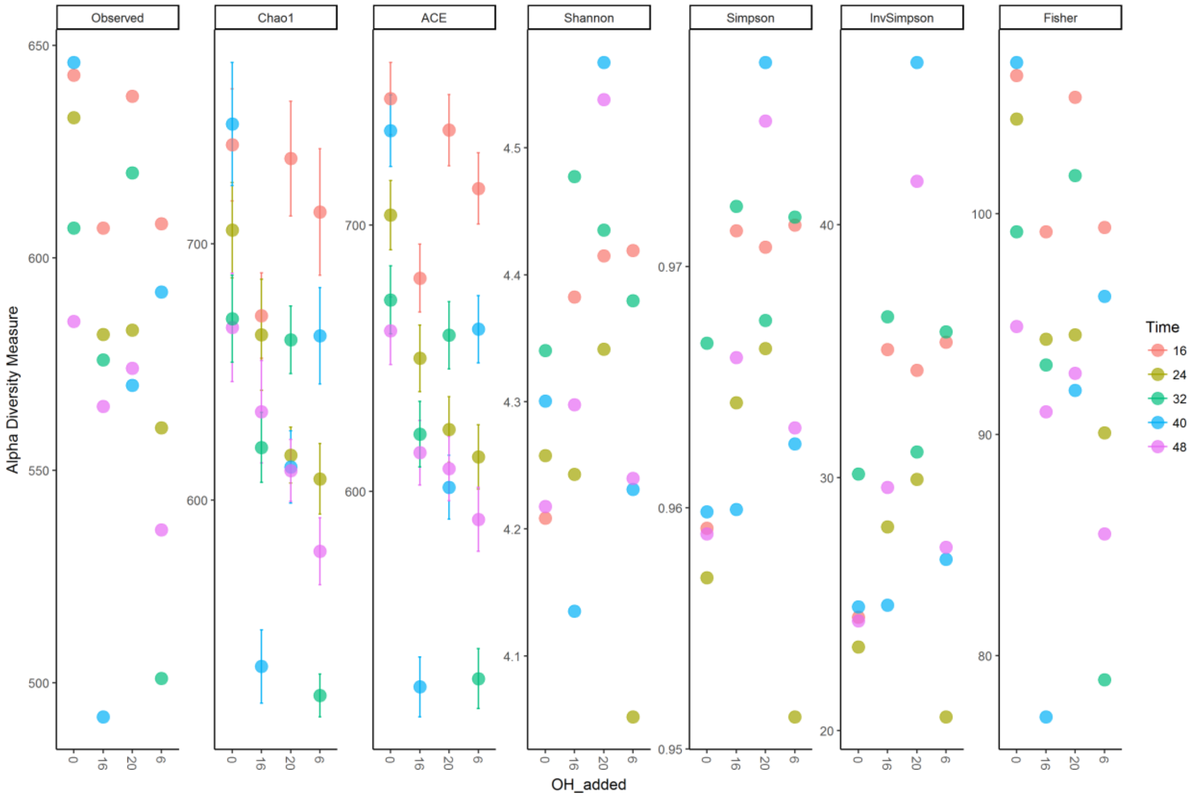

Supplementary Figure S12: Diversity measures for fungal OTUs. 0, 6, 16, and 20 "OH added" represents pH 4.7, 6.7, 8.3 and 8.8 respectively.

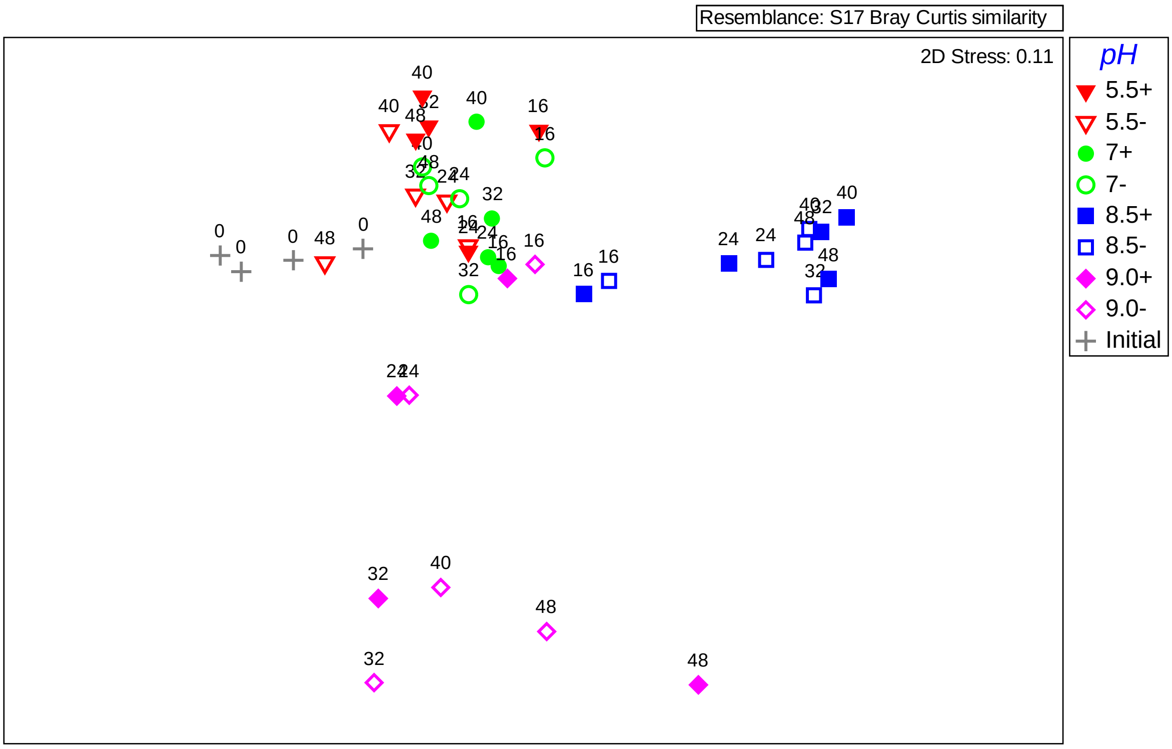

Supplementary Figure S13: MDS ordination of bacterial ARISA profiles showing bacterial community structural change with incubation time prior to DEA (denitrification enzyme activity) assays, at different pH values and + or - acetylene. Soils at pH 5.5 and 7 have very similar bacterial communities compared to the communities that develop in soil with pH altered to 8.5 and 9. Maximum denitrification occurred at pH 8.5 with approx. 24 hours pre-incubation prior to DEA-assay.

Microbial communities from the initial soil prior to any treatments being applied are also presented on the figure.

The soil was a Wakanui silt loam sampled under a no-tillage, arable cropping rotation at Lincoln. Soil pH was altered using KOH. Differing incubation times were used to investigate time needed for the community to induce the genetic machinery required for denitrification. Pre-incubation was conducted under anaerobic conditions with no added carbon sources aside from the native dissolved organic matter released via pH alteration.

Supplementary Table S1: Mineral N-chemistry, DOC and DON presenting changes during the 4 hour DEA period.

Decline during 4 h DEA assay	Pre-DEA Incubation time (h)	With Acetylene (N_2O-R blocked)				Without Acetylene (N_2O-R functional)			
		pH [KOH addition – $cmol_c\ kg^{-1}$]				pH [KOH addition – $cmol_c\ kg^{-1}$]			
		**4.7[0]**	**6.7[6]**	**8.3 [16]**	**8.7 [20]**	**4.7[0]**	**6.7[6]**	**8.3 [16]**	**8.7 [20]**
**NH_4^+ (mg kg^{-1})**	**16**	-0.1*	-2.5*	1.6	4.4	-0.3*	-0.6	1.5	5.0
	**24**	0.2*	-0.2*	0.5*	3.9	0.2*	0.7*	1.6*	5.5
	**32**	-0.1*	0.3*	9.0	4.9	0.0*	1.2	0.4*	5.7
	**40**	-0.1*	1.4	6.6	4.8	0.3*	2.3	3.4	5.0
	**48**	0.7	2.9*	2.4*	7.9	0.8*	2.1	4.6	3.6
**NO_3^- (mg kg^{-1})**	**16**	16.1	21.2	24.4	19.0	15.9	21.5	26.7	19.9
	**24**	16.4	22.7	41.3	21.5	16.6	22.0	35.5	22.9
	**32**	15.9	21.2	39.5	21.4	16.9	22.0	31.5	26.7
	**40**	15.4	21.5	36.0	25.5	16.2	19.0	31.2	25.6
	**48**	15.8	19.8	30.1	25.9	16.3	18.3	30.3	32.5
**DOC (mg kg^{-1})**	**16**	-8.5	32.1	736.5	-262.0*	-1.9*	35.8	976.2	945.0
	**24**	-0.7*	1.2*	944.9	1220.3*	-5.2*	60.9	893.6	1451.7*
	**32**	-0.2*	28.6*	707.2	965.6*	-0.7*	81.9	321.5*	949.9*
	**40**	8.2	57.0	453.2	164.2*	7.9	82.2	611.8	782.2*
	**48**	10.0	89.5	948.6	1420.9	9.6	67.6	941.9	1296.9
**DON (mg kg^{-1})**	**16**	1.7	4.9	78.8	-17.3*	-1.4*	2.7	67.3*	45.4*
	**24**	1.3*	-4.0*	46.0	72.5*	0.6*	4.8	63.8	117.4
	**32**	0.2*	5.3*	81.0	125.6	-1.1*	4.2	22.7*	21.6*
	**40**	3.8	4.2	90.6	15.3*	1.9*	6.4	65.0*	-5.8*
	**48**	-1.5*	10.0	26.0*	30.6*	2.1*	7.7	50.7	73.0*

*Difference not statistically significant at a 0.05 level based on Student's T-test between 'before' and 'after' DEA assay values. High variability occurs especially for DOC and DON.

Supplementary Table S2: ***Part A.*** Bacterial isolates grown on undiluted TSB-nitrate medium. Nitrate (NO_3^-) utilisation, ammonium (NH_4^+) produced and nitrous oxide (N_2O) emission data represents 48 hours from isolates grown in liquid TSB media compared to uninoculated controls.

Isolate Accessions:	Putative Identification via 16S rRNA gene:	No. of isolates	NO_3 utilised - range (mg L^{-1}) *(median)*	NH_4 produced - range (mg L^{-1}) *(median)*	N_2O emission - range (mg L^{-1}) *(median)*	CO_2 respiration range (g L^{-1}) *(median)*	GenBank accession numbers: SUB3915485	Notes: Possible N metabolism:
**150401_SRB**_1, 3, 6, 8, 9-12, 14, 15, 19, 20, 24, 26-28, 32a, 32b	*Bacillus sp.*	18	84 - 223 *(216)*	17 - 439 *(251)*	13 - 1222 *(431)*	10 - 45 *(30)*	**MH211**452, 456, 453, 457, 458, 455, 454, 459, 460, 451, 446, 449, 461, 444, 447, 450, 441, 462	Mostly DNRA, 1 N_2-fixer
**150401_SRB**_16, 25a, 33	*Paenibacillus sp.*	3	111 - 216 *(211)*	35 - 134 *(59)*	14 - 704 *(109)*	10 - 32 *(15)*	**MH211**436, 437,438	2 denitrifiers, 1 DNRA
**150401_SRB**_18	*Brevibacillus sp.*	1	187	84	101	16	**MH211**439	Denitrifier
**150401_SRB**_2, 5, 7, 13, 21*, 22, 23, 25B, 29, 30, 31	Not identified	11	12 - 223 *(36)*	-6 - 258 *(5)*	0 - 675 *(4)*	233 - 34 *(4)*		2 possible DNRA, 7 low/slow growth, 2 respiring with no N use.
	**Total**	**33**						

Supplementary Table S2: ***Part B.*** 1/10 diluted TSB-nitrate medium

Isolate Accessions:	Putative Identification via 16S rRNA gene:	No. of isolates	NO_3 utilised - range (mg L^{-1}) *(median)*	NH_4 produced - range (mg L^{-1}) *(median)*	N_2O emission - range (mg L^{-1}) *(median)*	CO_2 respiration range (g L^{-1}) *(median)*	GenBank accession numbers: SUB3915485	Notes: Possible N metabolism:
**150422_SRB**_1, 18*, 21, 27, 29, 31	*Bacillus sp.*	6	19 - 193 *(162)*	-9 - 536 *(121)*	0 - 426 *(179)*	-0.028 - 48 *(18)*	**MH211443,** 445, 442, 463, 448	2 isolates showed minimal growth.
**150422_SRB**_11, 14, 17, 19, 20, 24	*Acidovorax sp.*	6	192 - 193 *(193)*	11 - 43 *(21)*	0 - 2840 *(0)*	7 - 24 *(8)*	**MH211**427, 430, 429, 431, 428, 426	All denitrifers
**150422_SRB**_12	*Bosea sp.*	1	145	8	97	26	**MH211**435	Denitrifier
**150422_SRB**_36*	*Rhodanobacter sp.*	1	73	110	1071	21		DNRA
**150422_SRB**_37, 39a, 40	*Achromobacter sp.*	3	192 - 193 *(193)*	40 - 89 *(41)*	0 - 136 *(40)*	7 - 41 *(8)*	**MH211**433,434,432	All denitrifiers
**150422_SRB**_3, 4-10, 13, 15, 16, 22, 23, 25, 26, 28, 30a, 30b, 32-35, 38, 39b, 41, 43, 44	Not identified	27	-27 - 189 *(11)*	-12 - 486 *(11)*	0 - 610 *(2)*	0.05 - 57 *(14)*		Mostly low/slow growth isolates, 4 respiring with no N-use.
	**Total**	**44**						

*Sequences not of sufficient quality for submission to Genbank.

Supplementary Table S2: ***Part C.*** Isolates selected for genome sequencing.

Isolate Accessions:	Putative Identification via 16S rRNA gene:	No. of isolates	NO_3 utilised (mg L^{-1})	NH_4 produced (mg L^{-1})	N_2O emission (mg L^{-1})	CO_2 respiration (g L^{-1})	GenBank accession numbers: SUB3915485	Notes: Possible N metabolism:
**150401_SRB_08**	*Bacillus sp.*	1	223	267	1222	35	MH211457	DNRA, high N_2O, No N_2O-R?
**150401_SRB_28**	*Bacillus sp.*	1	84	439	87	45	MH211450	N_2-fixer
**150422_SRB_31**	*Bacillus sp.*	1	183	536	436	48	MH211448	DNRA
**150422_SRB_14**	*Acidovorax sp.*	1	193	11	2840	8	MH211430	Denitrifier, No N_2O-R?
**150422_SRB_17**	*Acidovorax sp.*	1	193	43	1	24	MH211429	Denitrifier
**150422_SRB_24**	*Acidovorax sp.*	1	192	19	35	7	MH211426	Denitrifier
